# Macroglia-derived thrombospondin 2 regulates alterations of presynaptic proteins of retinal neurons following elevated hydrostatic pressure

**DOI:** 10.1371/journal.pone.0185388

**Published:** 2017-09-27

**Authors:** Shuchao Wang, Tu Hu, Zhen Wang, Na Li, Lihong Zhou, Lvshuang Liao, Mi Wang, Libin Liao, Hui Wang, Leping Zeng, Chunling Fan, Hongkang Zhou, Kun Xiong, Jufang Huang, Dan Chen

**Affiliations:** Department of Anatomy and Neurobiology, Central South University School of Basic Medical Sciences, Changsha, Hunan, China; Tokai University, JAPAN

## Abstract

Many studies on retinal injury and repair following elevated intraocular pressure suggest that the survival ratio of retinal neurons has been improved by various measures. However, the visual function recovery is far lower than expected. The homeostasis of retinal synapses in the visual signal pathway is the key structural basis for the delivery of visual signals. Our previous studies found that complicated changes in the synaptic structure between retinal neurons occurred much earlier than obvious degeneration of retinal ganglion cells in rat retinae. The lack of consideration of these earlier retinal synaptic changes in the rescue strategy may be partly responsible for the limited visual function recovery with the types of protective methods for retinal neurons used following elevated intraocular pressure. Thus, research on the modulatory mechanisms of the synaptic changes after elevated intraocular pressure injury may give new light to visual function rescue. In this study, we found that thrombospondin 2, an important regulator of synaptogenesis in central nervous system development, was distributed in retinal macroglia cells, and its receptor α2δ-1 was in retinal neurons. Cell cultures including mixed retinal macroglia cells/neuron cultures and retinal neuron cultures were exposed to elevated hydrostatic pressure for 2 h. The expression levels of glial fibrillary acidic protein (the marker of activated macroglia cells), thrombospondin 2, α2δ-1 and presynaptic proteins were increased following elevated hydrostatic pressure in mixed cultures, but the expression levels of postsynaptic proteins were not changed. SiRNA targeting thrombospondin 2 could decrease the upregulation of presynaptic proteins induced by the elevated hydrostatic pressure. However, in retinal neuron cultures, elevated hydrostatic pressure did not affect the expression of presynaptic or postsynaptic proteins. Rather, the retinal neuron cultures with added recombinant thrombospondin 2 protein upregulated the level of presynaptic proteins. Finally, gabapentin decreased the expression of presynaptic proteins in mixed cultures by blocking the interaction of thrombospondin 2 and α2δ-1. Taken together, these results indicate that activated macroglia cells may participate in alterations of presynaptic proteins of retinal neurons following elevated hydrostatic pressure, and macroglia-derived thrombospondin 2 may modulate these changes via binding to its neuronal receptor α2δ-1.

## Introduction

Elevated intraocular pressure (IOP) is an important risk factor for the degeneration of retinal neurons and consequently causes visual deficits in some diseases such as glaucoma, diabetic retinopathy and age-related macular degeneration[1–4]. However, the degree of visual function recovery is far lower than expected when the survival ratio of neurons has already been improved by means of measures protecting neurons from injury[5, 6]. Thus, complementary strategies targeting different aspects to recover the damaged visual function after elevated IOP are necessary[7]. The retina is composed of five major neuron types: photoreceptor cells, interneurons (bipolar, horizontal, and amacrine cells) and retinal ganglion cells (RGCs), which form the synapses in the retinal outer and inner plexiform layers[8, 9]. Through these synapses, the visual signal is preliminarily integrated and transmitted to the brain[10]. Therefore, normal synaptic structure in the retina is vital for the maintenance of the function of visual processing. Accumulating evidence has shown that changes in synapses seem to precede neuronal death after nervous injury or disease[11, 12]. Park’s and our previous study also found that the presynaptic functional protein synaptophysin (SYN) exhibited spatiotemporal alterations immediately after acute elevated IOP, which were changed before RGC death[13, 14]. However, these changes were limited to the presynaptic components without being accompanied by alterations in postsynaptic elements, which means that no new and functional synapses were formed during this process[13]. As a result, the lack of consideration of the earlier abnormal retinal synaptic changes in the target and/or the time point of intervention for the previous rescue strategies may explain why the visual function is not effectively recovered when neural survival has been improved following many therapies. Now, the regulatory mechanisms of these synaptic changes after elevated IOP remain unclear and need to be explored.

Glia cells are the most abundant cells in the central nervous system (CNS)[15, 16]. Traditional views consider that glia cells provide substrates, energy metabolism and the physiological environment for neurons[17]. In recent years, glia cells have been thought to be active modulators in developing synaptogenesis [18, 19]. In many neuropathic and nervous injury states, glia cells can be activated, characterized by increased proliferation and glial fibrillary acidic protein (GFAP) expression[20, 21]. Our previous study also demonstrated that retinal synaptic changes are accompanied by activation of macroglia cells (astrocytes and Müller cells) following acute elevated IOP[22]. Meanwhile, the already increased expression of SYN was decreased when the activated macroglia cells were inhibited by fluorocitrate. This suggested that activated macroglia cells should play an important role in retinal synaptic changes induced by elevated IOP.

Several soluble factors, such as thrombospondins (TSPs), have been identified to mediate the glial influence on synaptogenesis in CNS development[23, 24]. The TSP family consists of five isoforms (TSP1/2/3/4/5)[25], which are detectable in the developing and adult CNS [26]. Most notably, TSP1 and TSP2 are the most common types in the nervous system[20, 27]. Our previous study found that the distributions of TSP1 and TSP2 in the rat retina are apparently distinct[28]. TSP2 is mainly present in macroglia cells and dramatically upregulated together with increased GFAP after elevated IOP. Meanwhile, the time frame of TSP2 upregulation is consistent with the period of retinal synaptic alterations. However, TSP1 is expressed in neurons and not significantly changed with IOP. These results suggest that TSP2 is the most likely factor to be secreted by activated macroglia cells and might be involved in synaptic alterations in retinal neurons after elevated IOP. α2δ-1 is a subunit of voltage-gated calcium channel and the neuronal TSP2 receptor identified by Eroglu and colleagues, which played an important role in CNS synaptogenesis [29]. Many other researchers also found that the expression of α2δ-1 was highly upregulated after peripheral and central nervous injury[30, 31]. Furthermore, in our previous study, we found that α2δ-1 was expressed in most retinal neuronal types including RGCs, horizontal cells, amacrine cells, and bipolar cells[28]. Meanwhile, the expression of α2δ-1 was increased after elevated IOP.

Based on our previous study and results from other researchers, we suppose that macroglia-derived TSP2 might modulate retinal presynaptic proteins changes by binding to its neuronal receptor α2δ-1. The research on the molecular mechanism of TSP2/α2δ-1 pathway may provide new insight in developing therapeutic strategies to alleviate the visual loss in retinal caused by elevated IOP.

## Materials and methods

### Mixed retinal macroglia /neuron cultures (mixed cultures)

All experimental procedures used in the present study were approved by the Institutional Review Board of the Third Xiangya Hospital of Central South University in accordance with the National Institutes of Health (NIH) guidelines for the Care and Use of Laboratory Animals. The information of animal research could check in the ARRIVE Guidelines Checklist.(See S1 File) Mixed cultures of retinal macroglia/ neuron cultures were prepared from 1-day-old neonatal Sprague-Dawley rat pups as described previously[32]. In brief, the eyes were removed aseptically and placed in sterile ice-cold PBS. The corneas were cut away, and the crystalline lens and pigment epithelium layer were removed, leaving the retinae. The retinae were placed in Dulbecco’s modified Eagle’s medium (DMEM) containing 0.02% papain and digested in a 5% CO_2_ incubator at 37°C for 18 min. Subsequently, the tissue was centrifuged and transferred to a new tube containing mixed culture medium (DMEM supplemented with 10% fetal bovine serum, 5% horse serum, 1% penicillin/streptomycin and 1% L-glutamine). Then, the tissue was triturated 50 times with a Pasteur pipette and filtered with a 70-mm nylon cell strainer. The suspension was centrifuged again, and the supernatant was removed for washing the tissue. After washing, the cells were re-suspended, counted with a hemocytometer and plated in T25 flasks and 35-mm dishes at a density of 2×10^5^ cells/ml. Cells were cultured in a 5% CO_2_ incubator at 37°C. Twelve hours (h) after plating, the medium was removed and replaced with fresh mixed culture medium. Every 2 days, half of the medium was changed with fresh neurobasal medium supplemented with B27 so that the concentration of the serum, which can provide energy for macroglia cells, was progressively reduced. The digital images of positively immunostained cells (in five randomly selected fields) were analyzed, and the cells were counted. The data were represented as the mean ± SEM from 3 independent experiments. The cells were used for experiments within 8–10 days after removal from the rat.

### Retinal neuron cultures

The method is basically consistent with mixed cultures as described above with the following reference [33]. First, to reach the proper neuron inoculum density, the cells were counted and plated at a density of 6×10^5^ cells/ml instead of 2×10^5^ cells/ml. Second, 4 h after plating, a time period in which the macroglia cells could not completely attach to the plates, the medium was removed so that most of the glia cells were also removed. Last, to further inhibit the proliferation of glia cells, 4 h after plating, the mixed culture medium was replaced with fresh neurobasal medium supplemented with B27. Therefore, the glia cells could not survive in this condition without serum.

### Pressure apparatus and experimental protocol

The retinal neuron cultures or mixed cultures were exposed to elevated hydrostatic pressure (EHP) as described previously[34]. The pressure variable chamber consisted of a pump, a pressure regulator, a value panel and a chamber and was placed in the normal incubator. A regulated mixture of 95% room air and 5% CO_2_ in the normal incubator was delivered to the chamber to achieve a constant high pressure. The pressure in the chamber was monitored by a mercurial sphygmomanometer directly connected to the outlet proximal to the chamber. The cultures were maintained under a constant high pressure of 100 mmHg for 2 h. The cells were then removed from the chamber, moved to a conventional culture incubator and allowed to recover for 2 h, 6 h, 12 h or 24 h.

### Immunofluorescence

The cells on 35-mm dishes were fixed for 20 min with 4% paraformaldehyde (PF) and washed three times for 5 min in ice-cold 0.01 M phosphate-buffered saline (PBS) solution. Subsequently, the cells were blocked for 1 h in blocking buffer that is PBS containing 5% normal bovine serum and 0.3% Triton X-100. The cultures were then incubated with combinations of the primary antibodies against the following targets: GFAP (1:200, Calbiochem, IF03L, Darmstadt, Germany), thrombospondin 2 (1:1000, Abcam, ab84469, Cambridge, UK), MAP2 (1:200, Sigma-Aldrich, M9942, St. Louis, MO, USA), α2δ-1 (1:200, Sigma-Aldrich, c5105, St. Louis, MO, USA), synapsin (1:500, Synaptic Systems, 122121, Goettingen, Germany), Homer-1b/c (1:200, SANTA, SC-20807, Dallas, USA), Gephyrin (1:500, Abcam, ab32206, Cambridge, UK) for one night at 4°C. On the next day, the cultures were shifted to room temperature for 30 min. Then, the cultures were washed 3 times as described above and incubated with Alexa-conjugated secondary antibodies (1:500, Jackson Immuno Research, West Grove, PA, USA) for 2 h. After washing three times in PBS, the cultures were covered with Vectashield mounting medium containing DAPI (Vector Laboratories, Burlingame, USA). The cells immunostained with the α2δ-1 antibody were not permeabilized with Triton-X to limit antibody staining to the cell surface[35]. For immunofluorescence intensity analysis, all the cell cultures were stained simultaneously under the same setting. Fluorescent images were taken at the same setting under the fluorescence microscope (Nikon, Tokyo, Japan). For synaptic puncta analysis, the puncta were quantified along 20 μm-long axon by a custom-written plug-in (Barry Wark) for the NIH image processing package Image J. The longest dendrite was identified as the axon. Puncta in 10 fields of neurons on three repeated coverslips were counted.

### Western blot

For Western blot analyses, the cell cultures were lysed in 100 μl RIPA containing 1% protease inhibitors (CWBIO Technology, Beijing, China). The cell extracts were incubated at 4°C for 45 min and then centrifuge at 12000g for 20 min at 4°C. The supernatant was collected carefully. The protein concentration was determined by BCA assay and 10 μg of each protein sample was loaded per lane. Proteins were separated by 4%–6% or 10% SDS-PAGE gel and transferred to nitrocellulose membrane (GE Healthcare, Little Chalfont, United Kingdom). The membranes were blocked with blocking buffer containing 5% nonfat milk in Tris-buffered saline with 0.1% Tween 20 (TBS-T) for 1 h or 3 h at room temperature and then incubated overnight at 4°C in blocking buffer containing primary antibodies: GFAP (1:300), TSP2 (1:1000), α2δ-1 (1:500), Synapsin (1:5000), SYN (1:2000, Sigma-Aldrich, s5768, St. Louis, MO, USA), PSD95 (1:5000, 3450s, Cell Signaling, Danvers, USA), Homer-1b/c (1:1000) or beta-tubulin (1:2000, Beyotime, AG019, Biotechnology, Haimen, China). Next day the cultures were shifted to room temperature for 30min. After washing 3 times, the membranes were incubated in blocking buffer containing HRP-conjugated anti-rabbit or anti-mouse IgG (1:1000, Beyotime) for 2 h at room temperature. After washing 3 times, the immunoreactive bands were visualized by low or high sensitivity chemiluminescence reagent (CWBIO Technology). Beta-tubulin was used as an internal reference control. Integrated density values of specific proteins were quantified using ImageJ software (ImageJ version 1; National Institutes of Health, USA) and normalized to the values of beta-tubulin.

### SiRNA approach

To determine whether TSP2 was required for the change in synaptic proteins after EHP, we used the small interfering RNA knockdown approach to partially reduce TSP2 expression. The siRNA kit against TSP2 was obtained from RIBO-Biology (Guang Zhou, China), and the transfection reagent was from MACS (Teterow, Germany). In companion control experiments, glia cells were transfected with the same amount of either a targeting control or a non-targeting control. Another group with transfection reagent alone was used as a normal control. The transfection protocol followed the MACS manufacturer’s instructions: First, the siRNA and transfection reagent were separately diluted in opti-MEM (Gibco, Grand Island, USA) for 5 min. Then, the diluted siRNA and transfection reagent solutions were mixed together for another 20 min for complex formation. Last, the cell medium was replaced with neurobasal medium, and the transfection complexes were added dropwise to the cultures. Four hours after transfection, the culture medium was replaced with normal neurobasal medium plus B27. Twenty-four hours after transfection, the cultures were collected for the next experiments. Knockdown of TSP2 was validated by Western blot.

### Drug application

Purified human recombinant TSP2 protein was purchased from R&D systems (1635-T2, Minnesota, USA) and gabapentin (GBP) from SANTA CRUZ (60142-96-3, Dallas, USA). All drugs were dissolved in sterile PBS as stock solutions and warmed to room temperature before performing experiments. TSP2, which was used at a concentration of 10 nM (~1.29 μg/ml), and gabapentin, which was used at a concentration of 32 μM (~5.47 μg/ml), were administered directly to cell cultures before high pressure treatment. The incubations were maintained for 14 h, including 2 h cultured in high pressure, and subsequently subjected to immunofluorescence and Western Blot.

### Statistical analysis

Figure panels were assembled by using Photoshop CC. The measurement data were presented as the mean ± SD. One-way analysis of variance and independent sample t-tests were used to analyze the data using GraphPad Prism 5 software (GraphPad Software Inc, San Diego, CA, USA). A value of P < 0.05 was considered statistically significant. The statistical graphs were created with GraphPad Prism 5 software.

## Results

### Culture compositions

**Fig 1:**
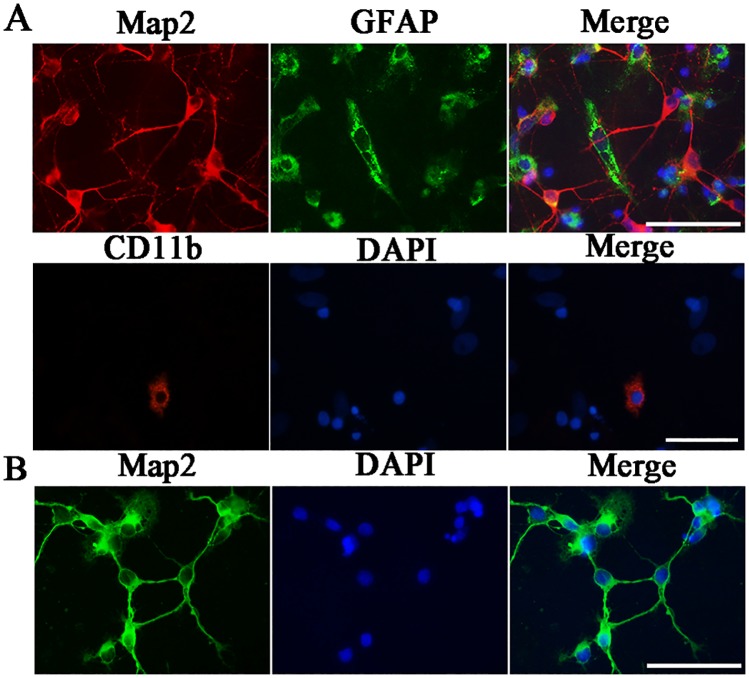
Cell compositions in the mixed cultures and retinal neuron cultures. (A) Double immunofluorescence Map2/GFAP, CD11b/DAPI staining in mixed cultures. (B) Double immunofluorescence Map2/DAPI staining in retinal neuron cultures. Scale bar = 50 μm.

The culture compositions were determined by cell counting after double immunofluorescence staining. Map2 and GFAP, and CD11b were used as markers of neurons, macroglia, and microglia cells, respectively, and the cell nuclei were labeled with DAPI. The results showed that the mixed cultures consisted of approximately (33.16±2.56) % neurons, (60.15±2.1) % macroglia cells and 4.37±3.6% microglia cells (Fig 1A). The retinal neuron cultures appeared to be (92.35±1.68) % pure as assessed by immunocytochemistry of Map2 (Fig 1B).

### Localization of TSP2 and α2δ-1

**Fig 2:**
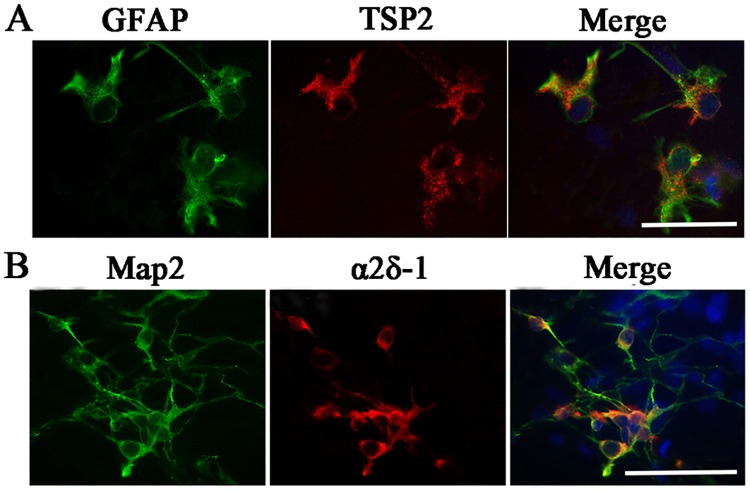
Immunofluorescence for the localization of TSP2 and α2δ-1. (A) Localization of TSP2 was identified by TSP2/GFAP co-staining. (B) Localization of α2δ-1 was identified by α2δ-1/Map2 co-staining. Scale bar = 50 μm.

Double immunofluorescence staining was performed, and TSP2 staining was co-localized with GFAP immunoreactivity (Fig 2A). α2δ-1 was strongly expressed in the retinal neuron bodies, and reduced staining was observed in dendrites visualized by co-localization with Map2, which is consistent with Farrell’s report[36] (Fig 2B).

### The expression levels of presynaptic proteins and GFAP were increased after EHP in mixed cultures

**Fig 3:**
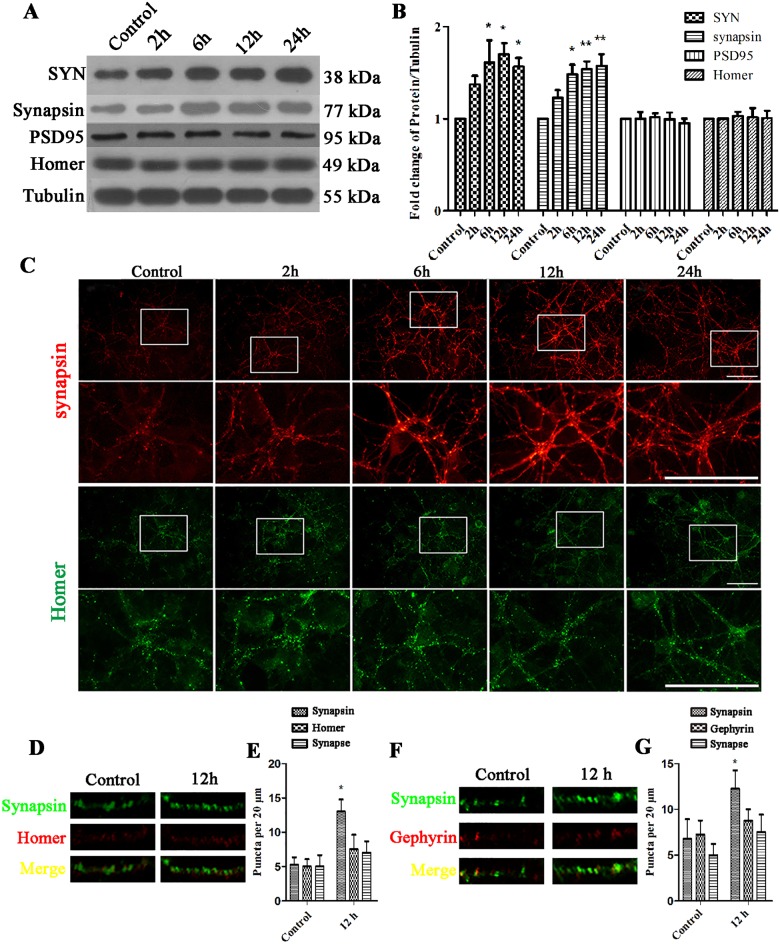
Expression of SYN, synapsin, Gephyrin, PSD95 and Homer following EHP in mixed cultures. Labels are as follows: Control group (Control); 2, 6, 12, and 24 h after EHP (2 h, 6 h, 12 h, and 24 h). (A) Western Blot of SYN, synapsin, PSD95 and Homer following EHP. (B) The statistical analysis of SYN, synapsin, PSD95 and Homer expression by Western Blot following EHP, * compared to Control, P<0.05; ** compared to Control, P<0.01. PSD95 and Homer expression show no statistically significant difference among groups (P>0.05). (C) Immunofluorescence staining of synapsin and Homer following EHP. Scale bar = 50 μm. The lower panels are the magnified images of the area in the rectangles of the upper panels. (D) Double immunofluorescence synapsin/Homer staining and their colocalization (synapse) in the dendrites per 20 μm. (E) Quantification of the number of synapsin, Homer and their colocalization puncta. * compared to Control, P<0.05. The number of Homer positive puncta and synapses show no statistically significant difference among groups (P>0.05) (F) Double immunofluorescence synapsin/Gephyrin staining and their colocalization in the dendrites per 20 μm. (G) Quantification of the number of synapsin, Gephyrin and their colocalization puncta. * compared to Control, P<0.05. The number of Gephyrin positive puncta and synapses show no statistically significant difference among groups (P>0.05).

First, we quantified the expression levels of the pre/postsynaptic proteins using Western Blot and immunofluorescence in the EHP groups (recovery time point for each group: 2 h, 6 h, 12 h and 24 h) after 2 h EHP in mixed cultures. From the results of the Western Blot, statistical analysis of the integrated density value (IDV) indicated that the expression levels of presynaptic SYN and synapsin were significantly increased 6 h, 12 h and 24 h (P<0.05) after EHP (Fig 3A and 3B). However, exposure of mixed cultures to EHP had no effect on the expression of postsynaptic PSD95 and Homer (Fig 3A and 3B). Immunofluorescence assay also showed that the immunofluorescence intensity of synapsin was increased, but the immunofluorescence intensity of homer was not changed (Fig 3C). We further stain the neuron with synapsin, Homer and Gephyrin and take the images at high magnification to show how the synapses formed after EHP injury. The immunostaining results showed that the number of synapsin positive puncta in the EHP groups were increased than that of the control group (Fig 3D, 3E, 3F and 3G), and the number of Homer (Fig 3D and 3E) and Gephyrin (Fig 3F and 3G) positive puncta were slightly increased but not significantly. These results showed that, in mixed cultures, EHP could increase the expression of presynaptic proteins but not postsynaptic proteins.

**Fig 4:**
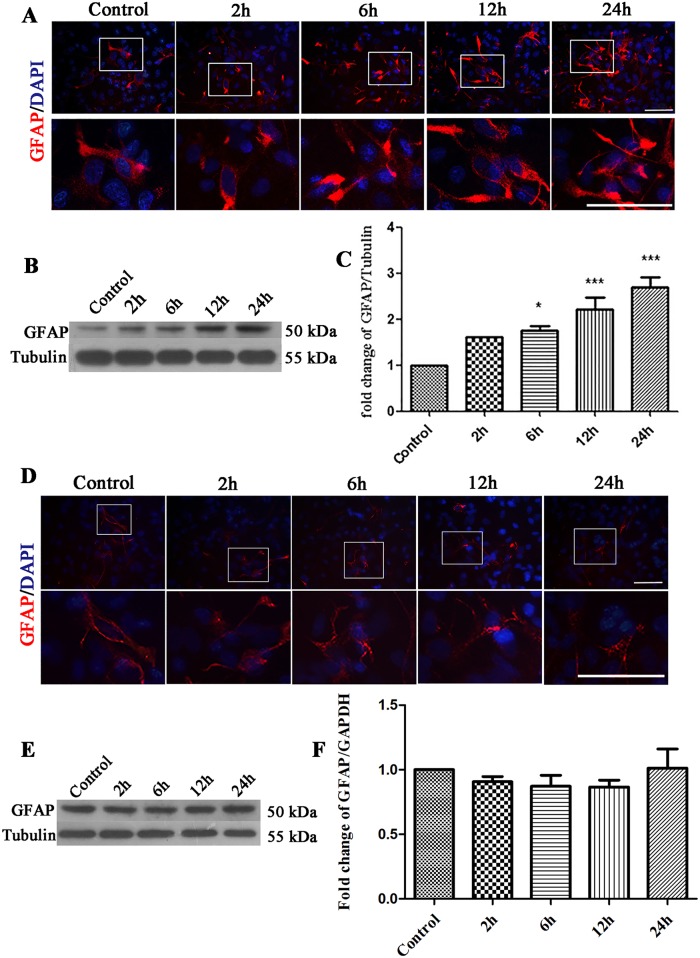
Expression of GFAP after EHP and in general during mixed cultures. Labels are as follows: Control group (Control); 2, 6, 12, and 24 h in general cultures (2 h, 6 h, 12 h, and 24 h). (A) Immunofluorescence staining of GFAP following EHP. Scale bar = 50 μm. The lower panels are the magnified images of the area in the rectangles of the upper panels. (B) Western Blot of GFAP following EHP. (C) The statistical analysis of GFAP expression by Western Blot following EHP, * compared to Control, P<0.05; ** compared to Control, P<0.01; *** compared to Control, P<0.005. PSD95 and Homer expression show no statistically significant difference among groups (P>0.05). (D) Immunofluorescence staining of GFAP. Scale bar = 50 μm. The lower panels are the magnified images of the area in the rectangles of the upper panels. (E) Western Blot of GFAP in general. (F) The statistical analysis of GFAP expression by Western Blot. GFAP expression show no statistically significant difference among groups (P>0.05).

Meanwhile, we observed a continuous increase in reactive macroglia cells indicated by increased proliferation and GFAP expression (Fig 4A). Statistical analysis of the Western Blot results showed that the expression of GFAP was significantly increased 6 h, 12 h and 24 h (P<0.05) after EHP (Fig 4B and 4C). To exclude the possible effect of macroglia cells which may become reactive during in vitro culture, we added the time-matched comparison that without EHP injury. From the western bolt and immunostaining results, the macroglia cells were not activated during in vitro culture, showed by unchanged GFAP expression (Fig 4D, 4E and 4F). Together, this and the above results indicated that retinal macroglia cells were activated when the expression of retinal presynaptic proteins was changed following EHP.

### The expression levels of synaptic proteins were not changed after EHP in retinal neuron cultures

**Fig 5:**
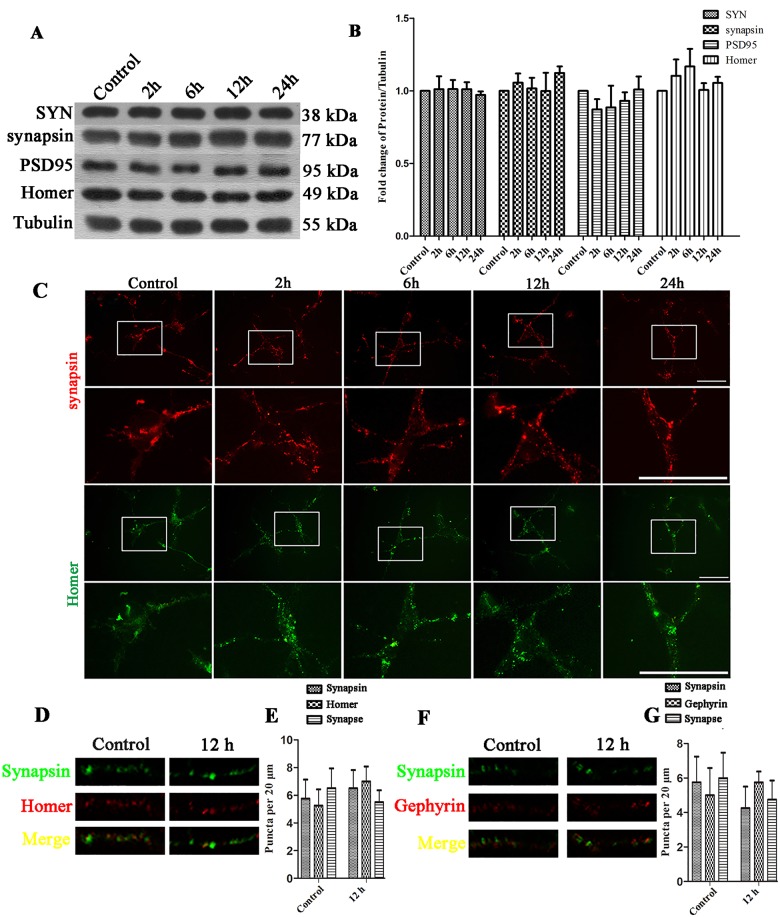
Expression of SYN, synapsin, Gephyrin, PSD95 and Homer following EHP in retinal neuron cultures. Labels are as follows: Control group (Control); 2, 6, 12, and 24 h after EHP (2 h, 6 h, 12 h, and 24 h). (A) Western Blot of SYN, synapsin, PSD95 and Homer following EHP. (B) The statistical analysis of SYN, synapsin, PSD95 and Homer protein expression following EHP, showing no statistically significant difference among groups (P>0.05). (C) Immunofluorescence staining of synapsin and Homer in retinal neurons following EHP. Scale bar = 50 μm. The lower panels are the magnified images of the area in the rectangles of the upper panels. (D) Double immunofluorescence synapsin/Homer staining and their colocalization in the dendrites per 20 μm. (E) Quantification of the number of synapsin, Homer and their colocalization puncta. The number of Synapsin, Homer positive puncta and synapses show no statistically significant difference among groups (P>0.05). (F) Double immunofluorescence synapsin/Gephyrin staining and their colocalization in the dendrites per 20 μm. (G) Quantification of the number of synapsin, Gephyrin and their colocalization puncta. The number of synapsin, Gephyrin positive puncta and synapses show no statistically significant difference among groups (P>0.05).

To investigate whether macroglia cells are necessary for the alterations in the presynaptic proteins after EHP, retinal neurons were cultured to eliminate the influence of macroglia cells. Retinal neuron cultures were exposed to EHP as described before. Statistical analysis of the Western Blot results indicated that EHP did not significantly change the expression of pre/postsynaptic proteins (Fig 5A and 5B). Moreover, the immunofluorescence assay showed that both the immunofluorescence intensity of synapsin and Homer were not changed (Fig 5C). Further images took at high magnification also indicated that no distinct changes in the number of pre/postsynaptic puncta between the EHP groups and control groups (Fig 5D, 5E, 5F and 5G). These results indicated that, in retinal neuron cultures without macroglia cells, EHP could not change the expression of synaptic proteins. Taken together, these results further suggested that macroglia cells may be involved in the change in retinal synapses following EHP.

### Upregulation of TSP2 and presynaptic proteins after EHP was downregulated in mixed cultures treated with TSP2 siRNA

**Fig 6:**
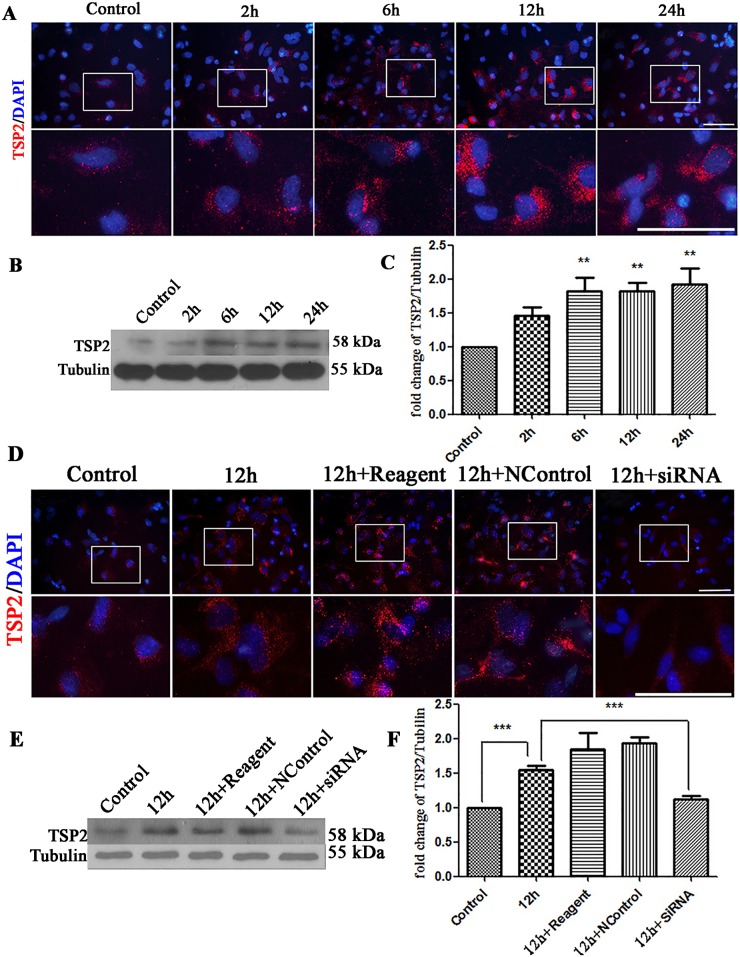
Expression of TSP2 under EHP and TSP2 siRNA silencing. Labels are as follows: Control group (Control); 2, 6, 12, and 24 h after EHP (2 h, 6 h, 12 h, and 24 h). (A) Immunofluorescence staining of TSP2 following EHP. The lower panels are the magnified images of the area in the rectangles of the upper panels. (B) Western Blot of TSP2 expression following EHP. (C) Statistical analysis of TSP2 expression following EHP, ** compared to Control, P<0.01. Scale bar = 50 μm. (D) Immunofluorescence staining of TSP2 following EHP and siRNA knockdown. Scale bar = 50 μm. The lower panels are the magnified images of the area in the rectangles of the upper panels. (E) Western Blot of TSP2 expression following EHP and siRNA knockdown. (F) The statistical analysis of TSP2 expression following EHP and TSP2 siRNA, * compared to Control, P<0.05. PSD95 and Homer expression show no statistically significant among groups (P>0.05).

Mixed cultures were exposed to EHP as described before, and the immunofluorescence intensity of TSP2 was enhanced in the injury groups compared with that in the control group (Fig 6A). Western Blot results indicated that EHP significantly increased the expression of TSP2 6 h, 12 h and 24 h after EHP (P<0.01) (Fig 6B and 6C).

**Fig 7:**
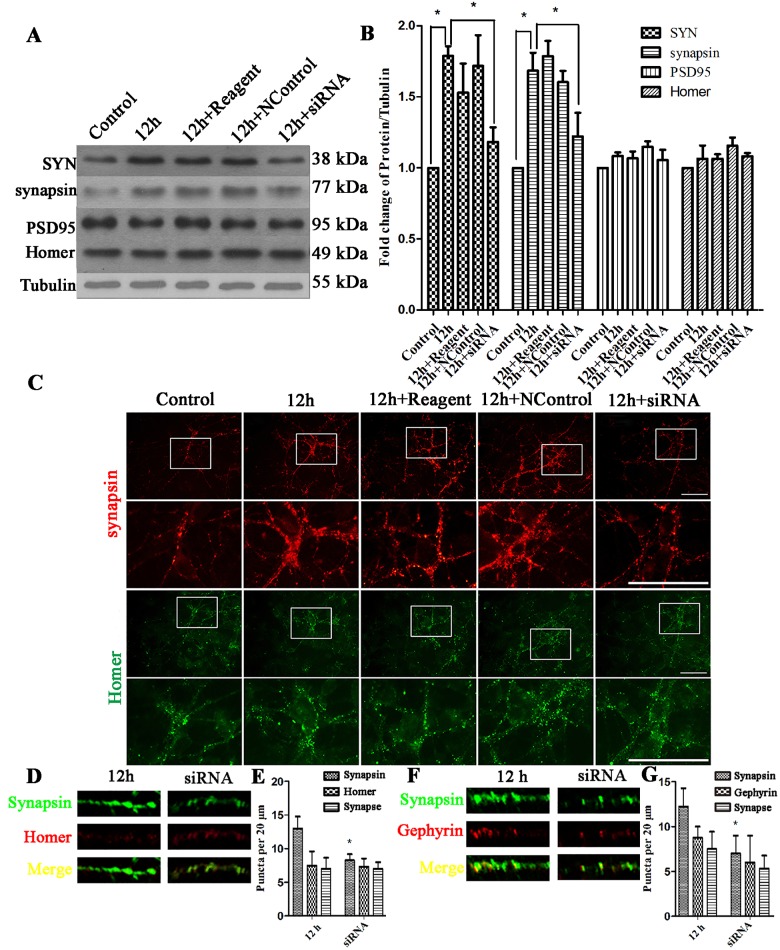
Expression of SYN, synapsin, Gephyrin, PSD95 and Homer under EHP and TSP2 siRNA silencing. Labels are as follows: Control group (Control), 12 h after EHP (12 h), Reagent + 12 h after EHP (12 h + Reagent), Non-targeting control + 12 h after EHP (12 h + NControl), siRNA + 12 h after EHP (12 h + siRNA). (A) Western Blot of SYN, synapsin, PSD95 and Homer expression following EHP and TSP2 siRNA. (B) The statistical analysis of SYN and synapsin expression following EHP and TSP2 siRNA, * compared to Control, P<0.05. PSD95 and Homer expression show no statistically significant among groups (P>0.05). (C) Immunofluorescence staining of synapsin and Homer following EHP and siRNA knockdown. Scale bar = 50 μm. The lower panels are the magnified images of the area in the rectangles of the upper panels. (D) Double immunofluorescence synapsin/Homer staining and their colocalization in the dendrites per 20 μm. (E) Quantification of the number of synapsin, Homer and their colocalization puncta. * compared to 12 h group, P<0.05. The number of Homer positive puncta and synapses show no statistically significant difference among groups (P>0.05) (F) Double immunofluorescence synapsin/Gephyrin staining and their colocalization in the dendrites per 20 μm. (G) Quantification of the number of synapsin, Gephyrin and their colocalization puncta. * compared to 12-h group, P<0.05. The number of Gephyrin positive puncta and synapses show no statistically significant difference among groups (P>0.05).

The synaptogenic properties of TSP2 and the relative increase in TSP2 and presynaptic proteins after EHP prompted us to research whether TSP2 is required for the upregulation of presynaptic proteins after EHP. To solve this, we examined whether silencing TSP2 with siRNA could prevent or diminish the level of upregulation of presynaptic or postsynaptic proteins. In the experiments above, we found that the expression of presynaptic proteins and TSP2 peaked at 6 h, 12 h, and 24 h after EHP, and we chose the middle time point (12 h) as our primary intervention time point. At first, we designed an artificial siRNA targeting TSP2 and transfected it into the mixed cultures. TSP2 was knocked down as demonstrated by the weaker immunoreactivity (Fig 6D) and blot (P<0.05) (Fig 6E and 6F). Then, in contrast with that in the 12 h EHP group, the Western Blot results showed that the expression levels of SYN and synapsin (P<0.05) in the siRNA group were lower than in the 12 h group (Fig 7A and 7B). While decreased expression of TSP2 did not cause any changes in the expression of postsynaptic proteins (Fig 7A and 7B). Besides, the enhanced immunostaining of synapsin was reduced in the siRNA group as showed by the weaker immunofluorescence (Fig 7C). Further analysis in the immunofluorescence assay showed that the number of synapsin positive puncta in the siRNA groups were decreased than in the 12 h group (Fig 7D, 7E, 7F and 7G), but the number of Homer (Fig 7D and 7E) and Gephyrin (Fig 7F and 7G) positive puncta were not significantly changed. Taken together, these results indicate that TSP2 is essential for the upregulation of presynaptic proteins in retinal neurons after EHP.

### Recombinant TSP2 protein upregulated the levels of presynaptic proteins after EHP in retinal neuron cultures

**Fig 8:**
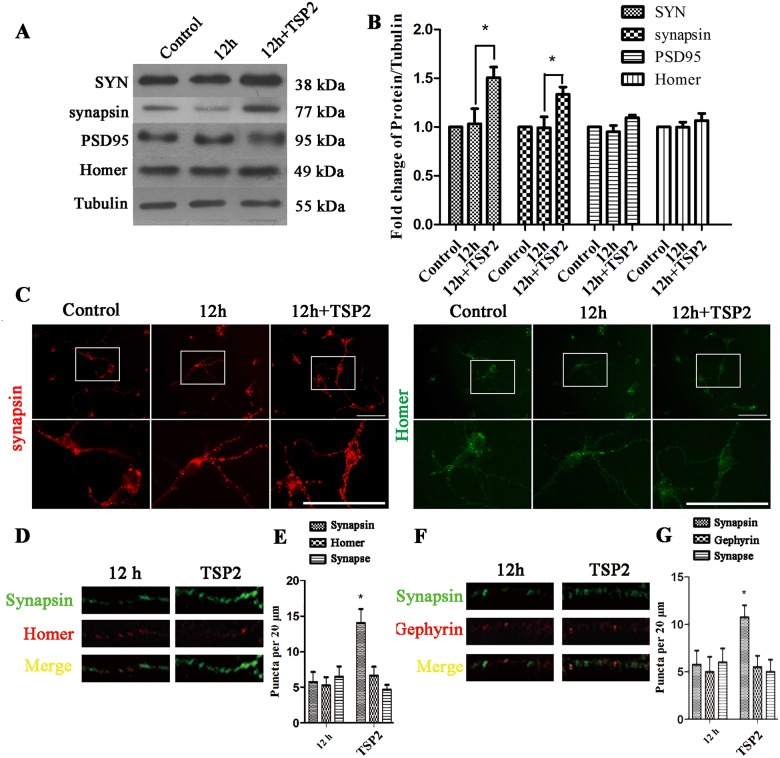
Expression of SYN, synapsin, Gephyrin, PSD95 and Homer under EHP and with the addition of recombinant TSP2 protein in retinal neuron cultures. Labels are as follows: Control group (Control), 12 h after EHP (12 h), recombinant TSP2 protein + 12 h after EHP (12 h + TSP2). (A) Western Blot of SYN, synapsin, PSD95 and Homer expression under EHP with the addition of recombinant TSP2 protein. (B) The statistical analysis of SYN and synapsin proteins following EHP and the addition of recombinant TSP2 protein, * compared to Control, P<0.05. PSD95 and Homer expression show no statistically significant difference among groups (P>0.05). (C) Immunofluorescence staining of synapsin and Homer following EHP and TSP2 application. Scale bar = 50 μm. The lower panels are the magnified images of the area in the rectangles of the upper panels. (D) Double immunofluorescence synapsin/Homer staining and their colocalization in the dendrites per 20 μm. (E) Quantification of the number of synapsin, Homer and their colocalization puncta. * compared to 12 h group, P<0.05. The number of Homer positive puncta and synapses show no statistically significant difference among groups (P>0.05) (F) Double immunofluorescence synapsin/Gephyrin staining and their colocalization in the dendrites per 20 μm. (G) Quantification of the number of synapsin, Gephyrin and their colocalization puncta. * compared to 12 h group, P<0.05. The number of Gephyrin positive puncta and synapses show no statistically significant difference among groups (P>0.05).

We also examined whether recombinant TSP2 protein directly contributed to the upregulation of the levels of presynaptic proteins after EHP. Accordingly, we applied recombinant TSP2 protein to retinal neuron cultures (without astrocytes; all TSP2 comes from the intervention) at the beginning of exposure to high pressure. The analysis was also conducted 12 h after EHP. The Western Blot results showed that the expression levels of SYN and synapsin (P<0.05) in the TSP2 group were higher than in the 12 h group, while the expression levels of PSD95 and Homer were maintained with or without treatment (Fig 8A and 8B). Direct exposure of retinal neurons to recombinant TSP2 protein for 12 h after EHP (TSP2 group) also resulted in an enhanced immunofluorescence (Fig 8C)and higher number of synapsin positive puncta (Fig 8D, 8E, 8F and 8G), but did not change the number of Homer (Fig 8D and 8E) and Gephyrin (Fig 8F and 8G) positive puncta. These data provided further evidence that TSP2 could directly upregulate the levels of presynaptic proteins after EHP in retinal neurons.

### Upregulated α2δ-1 was involved in synaptic changes after EHP in mixed cultures

**Fig 9:**
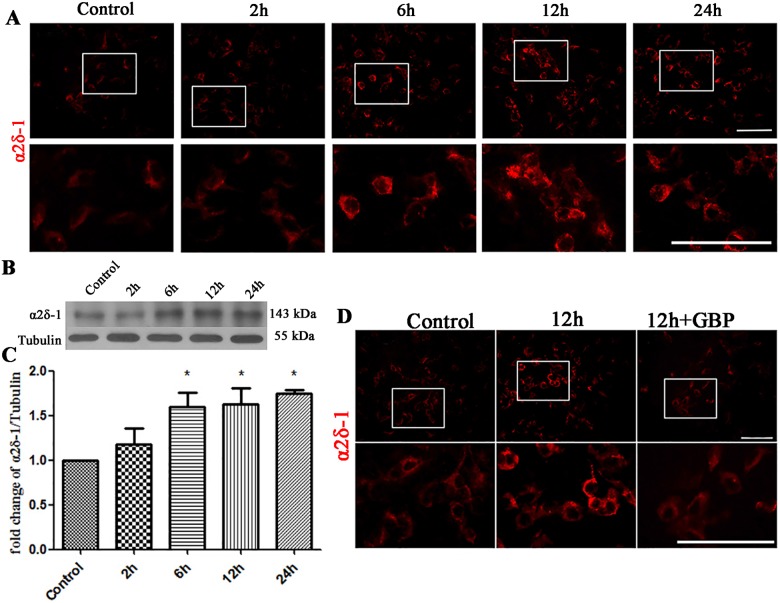
Expression of α2δ-1 under EHP and GBP application. Labels are as follows: Control group (Control); 2, 6, 12, and 24 h after EHP (2 h, 6 h, 12 h, and 24 h). (A) Immunofluorescence staining of α2δ-1 following EHP. Scale bar = 50 μm. The lower panels are the magnified images of the area in the rectangles of the upper panels. (B) Western Blot of α2δ-1 expression under EHP. (C) Statistical analysis of α2δ-1 expression levels under EHP, * compared to Control, P<0.05. (D) Immunofluorescence staining of α2δ-1, SYN, synapsin, PSD95 and Homer following EHP and GBP treatment. Scale bar = 50 μm. The lower panels are the magnified images of the area in the rectangles of the upper panels.

In this experiment, we investigated whether TSP2 modulated the presynaptic alterations of retinal neurons following EHP by binding to α2δ-1. Mixed cultures were exposed to EHP as described before. The immunofluorescence intensity of α2δ-1 was significantly enhanced after EHP compared with that in the normal control group (Fig 9A). The Western Blot results indicated that the expression levels of α2δ-1 6 h, 12 h and 24 h after EHP were increased compared with that in the control group (P<0.05) (Fig 9B and 9C).

Then, we used GBP to block the interaction of α2δ-1 with TSP2. GBP was added to the mixed cultures at the beginning of EHP, and the mixed cultures with GBP were incubated for another 12 h after EHP. We first tested whether GBP could decrease the expression of α2δ-1 on the cell surface. As was shown in the immunofluorescence assay, the immunofluorescence intensity of α2δ-1 in the GBP group was weaker than in the 12 h group (Fig 9D). This indicated that GBP could effectively decease the cell surface expression of α2δ-1 so that GBP could block the interaction of TSP2 with α2δ-1[35].

**Fig 10:**
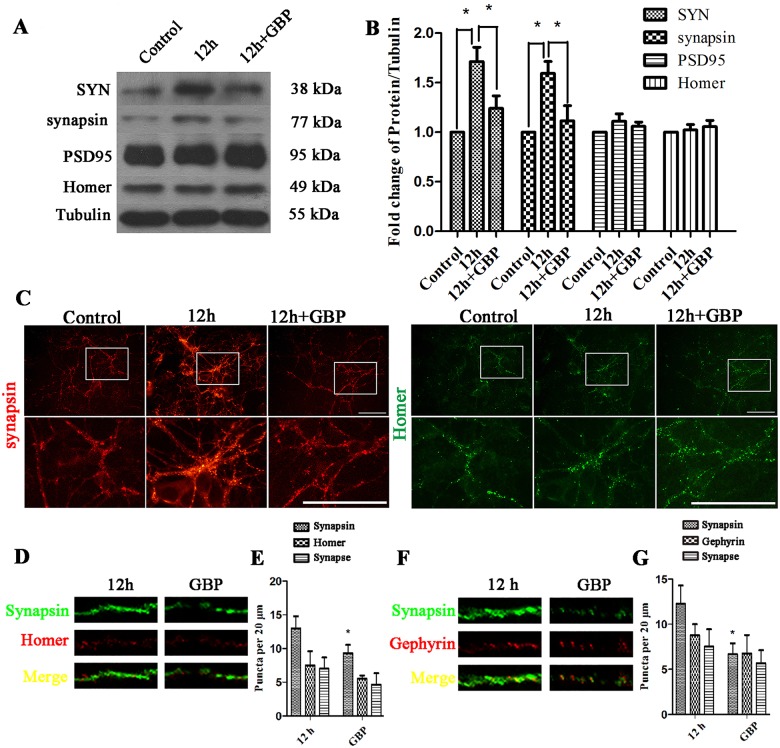
Expression of SYN, synapsin, Gephyrin, PSD95 and Homer under EHP and GBP treatment in mixed cultures. Labels are as follows: Control group (Control), 12 h after EHP (12 h), GBP + 12 h after EHP (12 h + GBP). (A) Western Blot of SYN, synapsin, PSD95 and Homer expression under EHP and GBP treatment. (B) Statistical analysis of SYN and synapsin expression levels under EHP and GBP treatment, * compared to Control, P<0.05. PSD95 and Homer expression show no statistically significant difference among groups (P>0.05). (C) Immunofluorescence staining of synapsin and Homer following EHP and GBP application. Scale bar = 50 μm. The lower panels are the magnified images of the area in the rectangles of the upper panels. (D) Double immunofluorescence synapsin/Homer staining and their colocalization puncta in the dendrites per 20 μm. (E) Quantification of the number of synapsin, Homer and their colocalization puncta. * compared to 12-h group, P<0.05. The number of Homer positive puncta and synapses show no statistically significant difference among groups (P>0.05) (F) Double immunofluorescence synapsin/Gephyrin staining and their colocalization in the dendrites per 20 μm. (G) Quantification of the number of synapsin, Gephyrin and their colocalization puncta. * compared to 12-h group, P<0.05. The number of Gephyrin positive puncta and synapses show no statistically significant difference among groups (P>0.05).

In the next experiment, we verified whether cell-surface α2δ-1 muting could reverse the upregulation of presynaptic proteins induced by EHP. The statistical analysis of the Western Blot results indicated that GBP treatment decreased the expression of SYN and synapsin in the GBP group compared with that in the 12-h group but did not change the expression of PSD95 and Homer (Fig 10A and 10B). The immunofluorescence results also indicated that the synapsin immunostaining was reduced after GBP treatment (Fig 10C). Furthermore, retinal neurons cultured with GBP resulted in deceased number of synapsin positive puncta (Fig 10D, 10E, 10F and 10G), but did not change the number of Homer (Fig 10D and 10E) and Gephyrin (Fig 10F and 10G) positive puncta. These results suggested that GBP could decrease the production of presynaptic proteins by blocking the interaction of α2δ-1 with TSP2 in mixed cultures.

## Discussion

Many reports have demonstrated that acute or continuous elevation of IOP could lead to retinal neuron death and visual deficits[1]. Cultured cells exposed to elevated pressure are commonly used in neuronal death models induced by elevation of IOP[37]. In our study, the open cycling air pressure culture system, which could set defined pressure values as required, was applied to induce high pressure and injure the cultured cells to mimic elevated IOP injury. Meanwhile, we used SYN, synapsin[26, 38], Gephyrin[39], PSD95 and Homer[38, 40], the popular markers of synaptic density, to observe synaptic changes in retinal neurons. We found that the expression levels of presynaptic SYN and synapsin in mixed cultures were increased after EHP. However, EHP did not change the expression of postsynaptic markers PSD95 and Home, as well as Gephyrin. We also found that GFAP immunoreactivity was increased, which indicated that macroglia cells were activated together with the presynaptic changes after EHP. At the same time, TSP2 and α2δ-1 protein levels also increased in mixed cultures after exposure to EHP. Thus, activated macroglia cells and the expression change of TSP2, α2δ-1 and presynaptic proteins could be triggered in concert with an associated event.

Glia cells have been shown to be able to be activated in neuropathic conditions, and the activated glia cells may release reactive factors to promote synaptogenesis in the developed nervous system. Then, the reactive synaptogenesis may result in various nervous system diseases [15, 20, 21]. Lo[41] found that, after neonatal unilateral transection of the infraorbital branch of the trigeminal nerve, glia cells were activated, demonstrated by cell proliferation and upregulation of GFAP, and accompanied by reactive synaptogenesis in the nerve projection zone. Meanwhile, reactive synaptogenesis was blocked when sodium fluoroacetate blocked the activated glia cell function. Consistent with this report, we found that retinal macroglia cells were activated after EHP in mixed cultures. Meanwhile, the expression levels of presynaptic SYN and synapsin in mixed cultures were increased, but the expression levels of postsynaptic proteins were not changed. Next, we removed the macroglia cells from the mixed cultures and found that retinal neuron cultures exposed to EHP did not exhibit a change in the expression of these presynaptic proteins. This was consistent with Crawford’s report that glia cell deprivation with 4% paraformaldehyde/0.2% glutaraldehyde mediated hippocampal presynaptic plasticity[42]. That result indicates that the increased expression of presynaptic proteins, without the accompanying changes in the expression of postsynaptic proteins, cannot form intact and functional synapses after EHP. Taken together, these results indicated that the activated macroglia cells play important roles in the changing process of synaptic proteins in retinal neurons induced by EHP. Now, it is possible to consider therapeutic strategies that target macroglia cells for the treatment of diseases induced by EHP. However, inhibition of macroglia cells, which play important roles in the nervous system, could also lead to other neurodegenerative diseases and injuries. Therefore, it is reasonable and important to discover the mechanisms of presynaptic plasticity induced by activated macroglia cells after EHP.

TSPs have been proven to be a key factor secreted by glia cells to promote synapse formation in the developing central and peripheral nervous system[24, 38]. Further research has demonstrated that following some nervous diseases or injury, such as stroke, hepatic encephalopathy, cerebral ischemia and hemorrhage, glia cells can be activated and re-express TSPs, which participate in the regulation of neuronal plasticity[43–45]. Crosby[46] found that increased expression of TSPs in the spinal cord following injury contributes to abnormal synaptogenesis and leads to neuropathic pain. Blocking TSP expression with antisense oligonucleotides can inhibit dorsal horn synaptogenesis and then attenuate injury-induced allodynia. In our prior study, we found that TSP2 was mainly located in retinal macroglia cells and sharply increased in vivo after EHP. The distribution and expression change of the TSP2 protein suggested that TSP2 might be the key molecule secreted by macroglia cells that may modulate synaptic changes after EHP. To prove the speculation above, we first utilized siRNA to silence TSP2 expression at the time when its protein level was upregulated by EHP in mixed cultures. In accordance with our prediction, we observed a significant decrease in both SYN and synapsin expression. To directly confirm that the TSP2 protein has an effect on the change in presynaptic protein expression after EHP, we added recombinant TSP2 protein to retinal neuron cultures. We found that exposure of neurons to the TSP2 protein could lead to increased expression of SYN and synapsin proteins. These results indicated that TSP2 may be secreted by macroglia cells and participate presynaptic proteins changes induced by EHP. Except for TSP2 secreted by reactive macroglia cells, recent studies demonstrated that TSP4 may also generated by reactive astrocytes and contribute to synaptogenesis after injury[47, 48]. Therefore, clearly further studies are needed to explore the synaptogenic roles of different increased TSPs after injury. It was also worthy to note that the microglia cells are also the source of TSP2[20]. The relatively small percentage of microglia cells is approximately 4% in retinal cell cultures. The evidences to recognize the important role of reactive macroglia cells in the synaptogenesis are growing in recent studies[49]. So that, we considered that macroglia cells (mainly astrocytes and Muller cells, rather than microglia cells) were the major responder cells in presynaptic protein changes in retinal neurons following EHP. Anyway, these results suggest that suppressing the upregulation of TSP2 may be a good way to inhibit the change in presynaptic proteins induced by EHP.

Eroglu identified that TSPs promote synapse formation by interacting with the neuronal receptor α2δ-1[29]. Andresen[50] also found that in an experimental model, following neonatal freeze-lesion, the expression levels of both TSP and α2δ-1 were transiently increased, resulting in synaptogenesis and cortical hyperexcitability. Treatment with GBP, a widely used clinical pharmaceutical drug that is used for treatment of pain, anxiety and epilepsy, could disrupt the interaction of TSP with α2δ-1 and would prevent the formation of epileptic activity[30, 51]. Taken together, we hypothesized that α2δ-1 may be the receptor of TSP2 involved in the presynaptic alterations in retinal neurons following EHP. Meanwhile, treatment with GBP might be one way to inhibit the significant change in presynaptic protein expression in the retina after elevated IOP. Therefore, in this study, we discovered that α2δ-1 was localized to retinal neurons (strongly immunostained in soma and weakly immunostained in dendrites) in vitro, which was the same in vivo. Then, in accordance with others reports, we found a significant increase in α2δ-1 in immunoreactivity after EHP, which coincided with TSP2 upregulation and presynaptic changes, suggesting that α2δ-1 may contribute to the changes in retinal synaptic plasticity after EHP. Next, we found that pretreatment with GBP, the inhibitor of the interaction of TSP2 with α2δ-1, could reduce the increase in presynaptic SYN and synapsin induced by EHP. Thus, our results indicated that α2δ-1 may be the receptor for TSP2 involved in the retinal synaptic changes after EHP. Furthermore, using GBP may contribute to the prevention of the significant change in presynaptic protein expression by inhibiting the interaction of TSP2 with α2δ-1 after EHP.

In the current study, we observed the upregulation of presynaptic proteins without the accompanying changes in postsynaptic proteins in primary mixed cultures exposed to EHP for 2 h. That means that EHP only induced presynaptic protein changes, but no intact and functional synapses were formed[13, 22]. However, protein synthesis function is highly dependent on mitochondrial energy production, and abnormal synthesis function may lead to energy deficits in cells and increased apoptosis[52]. Therefore, this presynaptic change process may place a burden on the energy-producing machinery of neurons and accelerate the processes of synaptic disruption and neuronal death[43, 53]. Studying this regulatory mechanism of presynaptic changes and looking for targets to inhibit this process may be able to save this ineffective energy consumption and eventually lead to new ideas for retinal protection. The current experiments first confirmed that retinal macroglia activation after EHP could modulate these synaptic alterations in retinal neurons and, next, determined that TSP2 secreted by macroglia cells in the presence of environmental stress induced during the course of EHP might regulate synaptic protein alterations by binding to its neuronal receptor α2δ-1 in primary mixed cultures. This regulatory pathway may represent attractive targets for pharmacological intervention and require the profound understanding of their distribution and role in the nervous system to develop targeted therapies for diseases and injuries[54]. Therefore, it is reasonable to expect that strategies targeting the macroglia-TSP2-α2δ-1 pathway to regulate presynaptic changes of retinal neurons after EHP may contribute to the recovery of visual function. Also, we suspect that another way to protect the visual pathway is to stimulate postsynaptic changes, such as those induced by glypicans, which can arouse the response of the postsynaptic components in response to the presynaptic synaptogenesis events[48,49]. Besides the presynaptic mechanism of TSP2/α2δ-1 pathway, we speculate macroglia might play a key role in a complementary mechanism of postsynaptic changes of retinal neurons after EHP, which requires further experimental exploration.

## Conclusion

This study demonstrated that retinal macroglia cells were activated in vitro after EHP, resulting in presynaptic alterations without the accompanying changes in postsynaptic proteins in retinal neurons. We next determined that TSP2 secreted by macroglia cells after EHP injury might regulate the presynaptic protein alteration by binding to its neuronal receptor α2δ-1. These results suggest that targeting the macroglia cell-TSP2-α2δ-1 pathway may contribute to the recovery of visual function after elevated IOP.

## Supporting information

S1 File ARRIVE guidelines checklist. (DOCX)

S2 File Certificate. (PDF)

S3 File Supporting information. (DOCX)
