# Supplementary material for: Macroglia-derived thrombospondin 2 regulates alterations of presynaptic proteins of retinal neurons following elevated hydrostatic pressure
Source: PLoS One. 2017 Sep 27;12(9):e0185388. doi: 10.1371/journal.pone.0185388 (PMC5617560; doi:10.1371/journal.pone.0185388)

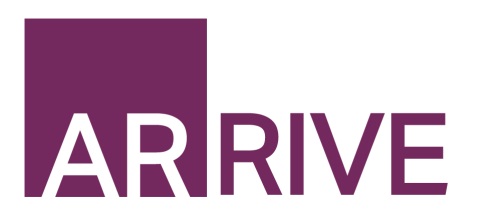


The ARRIVE Guidelines Checklist

Animal Research: Reporting In Vivo Experiments

Carol Kilkenny^1^, William J Browne^2^, Innes C Cuthill^3^, Michael Emerson^4^ and Douglas G Altman^5^

*^1^The National Centre for the Replacement, Refinement and Reduction of Animals in Research, London, UK, ^2^School of Veterinary Science, University of Bristol, Bristol, UK, ^3^School of Biological Sciences, University of Bristol, Bristol, UK, ^4^National Heart and Lung Institute, Imperial College London, UK, ^5^Centre for Statistics in Medicine, University of Oxford, Oxford, UK.*

|  | | ITEM | RECOMMENDATION | Section/ Paragraph |
| --- | --- | --- | --- | --- |
| 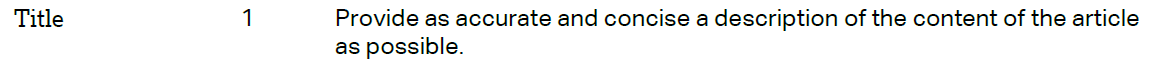 | | | Title |  |
| 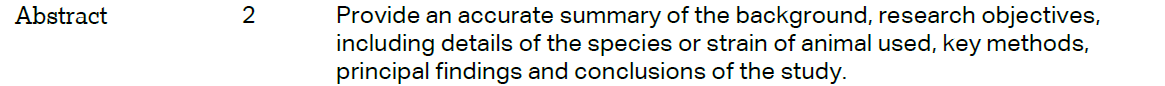 | | | Abstract |  |
| INTRODUCTION | | |  |  |
| 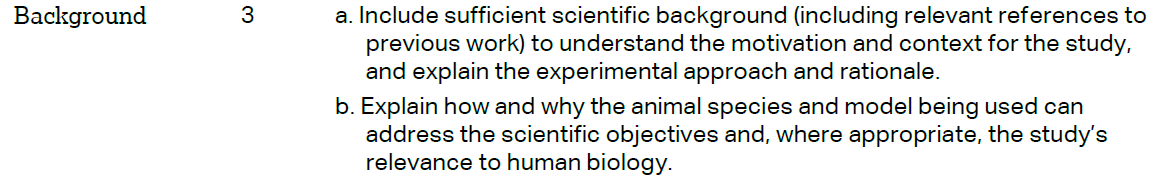 | | | Paragraph1-4  Discussion  Paragraph1 |  |
| 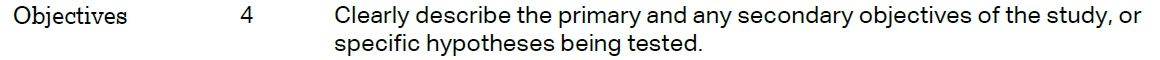 | | | Paragraph4 |  |
| METHODS | | |  |  |
| 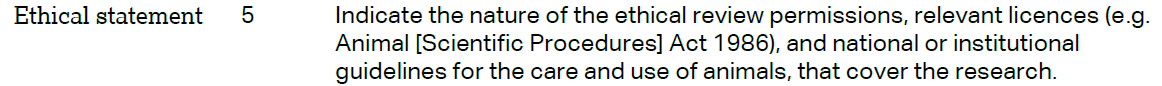 | | | Paragraph1 |  |
| 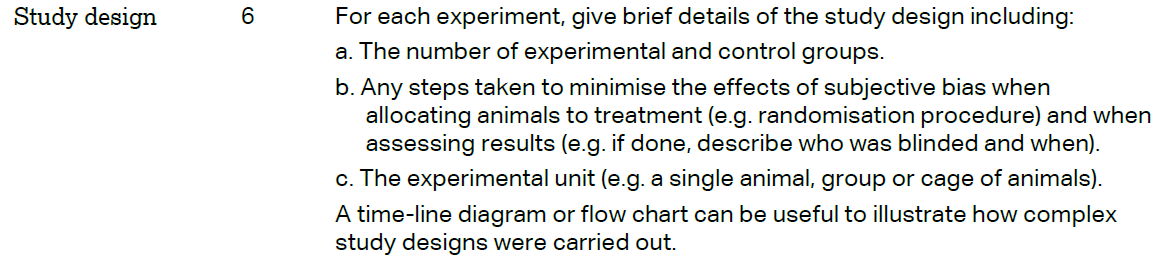 | | | Paragraph1, 3 |  |
| 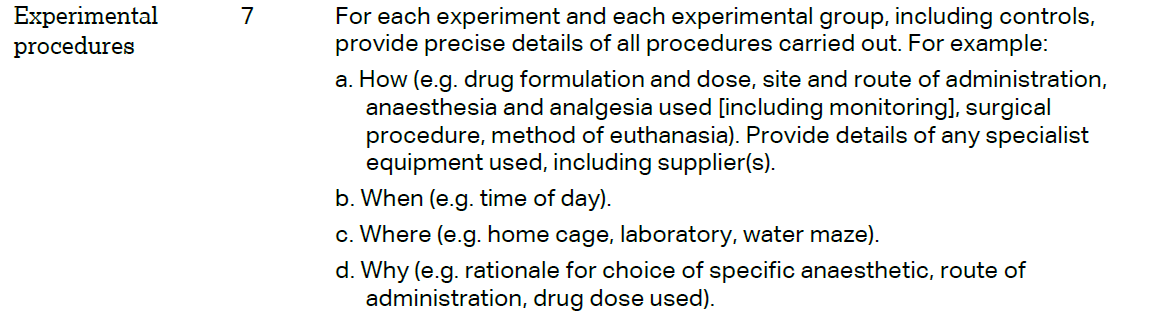 | | | Paragraph1 |  |
| 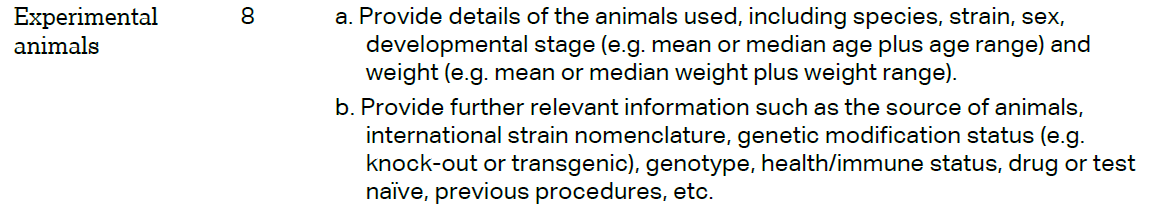 | | | Paragraph1 |  |

The ARRIVE guidelines. Originally published in *PLoS Biology*, June 2010^1^

| 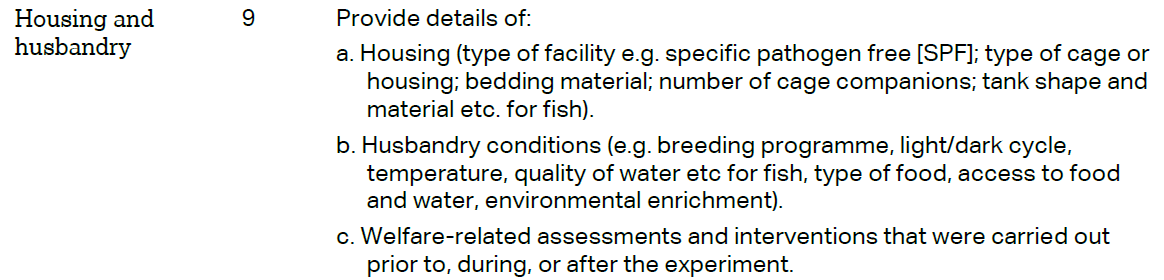 | / | |
| --- | --- | --- |
| 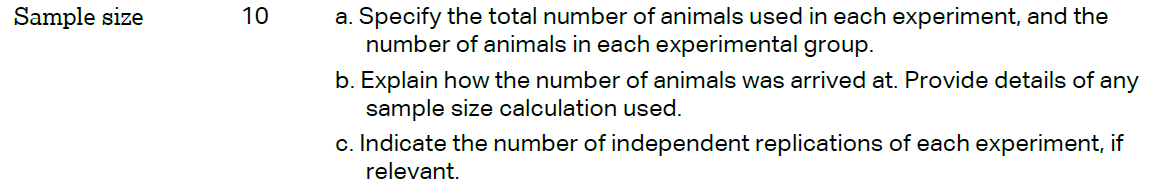 | Paragraph1, 3 | |
| 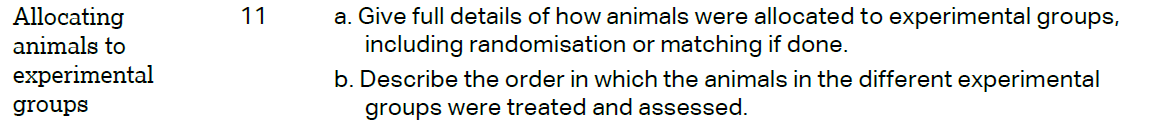 | / | |
| 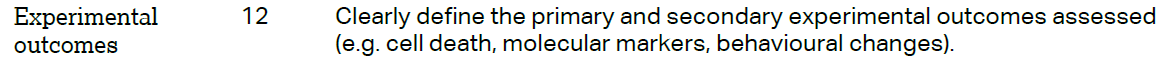 | / | |
| 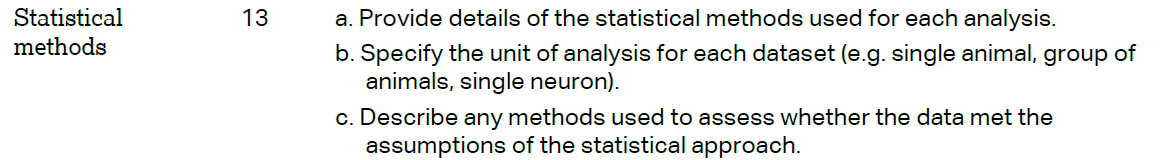 | Paragraph8 | |
| RESULTS |  | |
| 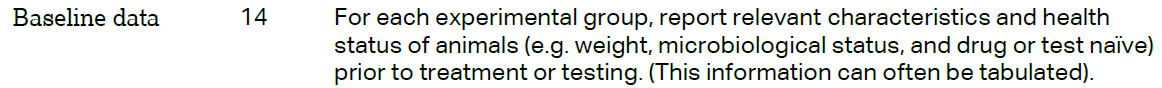 | / | |
| 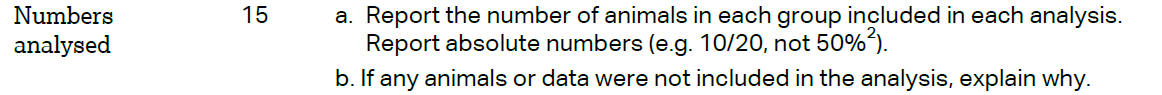 | / | |
| 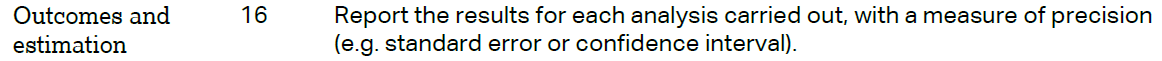 | / | |
| 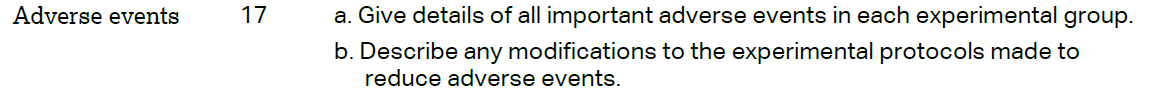 | / | |
| DISCUSSION |  | |
| 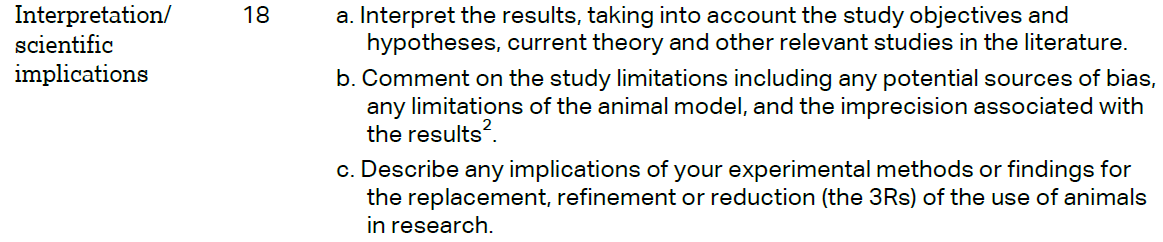 | Throughout  Paragraph 5  / | |
| 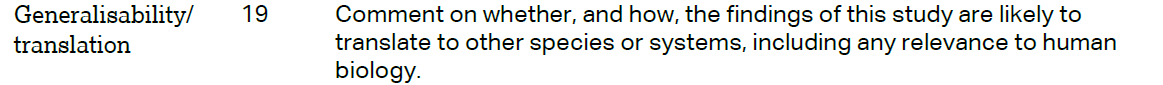 | Paragraph1, 3, 5 | |
| 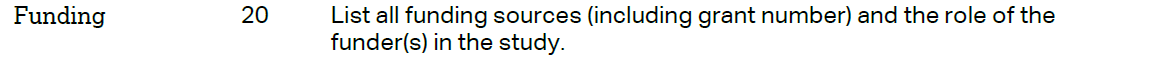 | | Financial Disclosure |


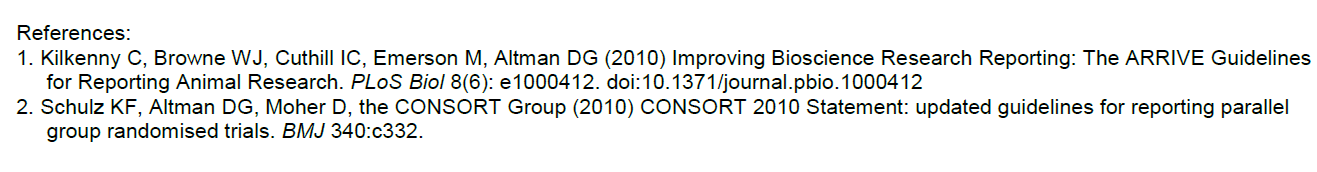

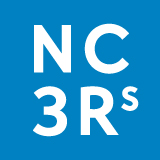

Supplement: S1 File — (DOCX) [file pone.0185388.s001.docx]
