# Supplementary material for: Macroglia-derived thrombospondin 2 regulates alterations of presynaptic proteins of retinal neurons following elevated hydrostatic pressure
Source: PLoS One. 2017 Sep 27;12(9):e0185388. doi: 10.1371/journal.pone.0185388 (PMC5617560; doi:10.1371/journal.pone.0185388)
Supplement: S3 File — (DOCX) [file pone.0185388.s003.docx]

Dear Editors:

We would like to submit the enclosed manuscript entitled “Macroglia-derived thrombospondin 2 regulates alterations of presynaptic proteins of retinal neurons following elevated hydrostatic pressure”.

　In the past years, our laboratory has investigated the possible [pathological](javascript:void(0);) mechanisms of eye diseases characterized by elevated intraocular pressure (IOP). Although the survival ratio of retinal neurons has been improved by certain measures, the visual function recovery is far lower than expected. A series of reports and our previous studies (Dan Chen, Acta Histochem. Cytochem.2008; Jufang Huang, Neural regeneration research, 2012; Jufang Huang, Experimental eye research, 2013; Lihong Zhou, Acta Histochem. Cytochem, 2013; Lihong Zhou, Neural regeneration research, 2014.) have indicated that complicated changes in the synaptic structure between retinal neurons occur in rat retinae after IOP.

In this research article, we report a new unexplored function for thrombospondin 2 in the control of synaptic changes between retinal neurons after elevated hydrostatic pressure (EHP). Activated macroglia cells participate in alterations of presynaptic proteins of retinal neurons following EHP, and macroglia-derived TSP2 may modulate these changes via binding to its neuronal receptor α2δ-1. Our research observations could have more implications in elevated IOP diseases and injuries and may provide new attractive interventional targets for visual function recovery.

**Abstract**

Many studies on retinal injury and repair following elevated intraocular pressure suggest that the survival ratio of retinal neurons has been improved by various measures. However, the visual function recovery is far lower than expected. The homeostasis of retinal synapses in the visual signal pathway is the key structural basis for the delivery of visual signals. Our previous studies found that complicated changes in the synaptic structure between retinal neurons occurred much earlier than obvious degeneration of retinal ganglion cells in rat retinae. The lack of consideration of these earlier retinal synaptic changes in the rescue strategy may be partly responsible for the limited visual function recovery with the types of protective methods for retinal neurons used following elevated intraocular pressure. Thus, research on the modulatory mechanisms of the synaptic changes after elevated intraocular pressure injury may give new light to visual function rescue. In this study, we found that thrombospondin 2, an important regulator of synaptogenesis in central nervous system development, was distributed in retinal macroglia cells, and its receptor α2δ-1 was in retinal neurons. Cell cultures including mixed retinal macroglia cells/neuron cultures and retinal neuron cultures were exposed to elevated hydrostatic pressure for 2 h. The expression levels of glial fibrillary acidic protein (the marker of activated macroglia cells), thrombospondin 2, α2δ-1 and presynaptic proteins were increased following elevated hydrostatic pressure in mixed cultures, but the expression levels of postsynaptic proteins were not changed. SiRNA targeting thrombospondin 2 could decrease the upregulation of presynaptic proteins induced by the elevated hydrostatic pressure. However, in retinal neuron cultures, elevated hydrostatic pressure did not affect the expression of presynaptic or postsynaptic proteins. Rather, the retinal neuron cultures with added recombinant thrombospondin 2 protein upregulated the level of presynaptic proteins. Finally, gabapentin decreased the expression of presynaptic proteins in mixed cultures by blocking the interaction of TSP2 and α2δ-1. Taken together, these results indicate that activated macroglia cells participate in alterations of presynaptic proteins of retinal neurons following elevated hydrostatic pressure, and macroglia-derived TSP2 may modulate these changes via binding to its neuronal receptor α2δ-1.

**Keywords:** macroglia cell; thrombospondin 2; α2δ-1; elevated hydrostatic pressure; retina; synapse

**Introduction**

Elevated intraocular pressure (IOP) is an important risk factor for the degeneration of retinal neurons and consequently causes visual deficits in some diseases such as glaucoma, diabetic retinopathy and age-related macular degeneration[[1-4](#_ENREF_1)]. However, the degree of visual function recovery is far lower than expected when the survival ratio of neurons has already been improved by means of measures protecting neurons from injury[[5](#_ENREF_5), [6](#_ENREF_6)]. Thus, complementary strategies targeting different aspects to recover the damaged visual function after elevated IOP are necessary[[7](#_ENREF_7)]. The retina is composed of five major neuron types: photoreceptor cells, interneurons (bipolar, horizontal, and amacrine cells) and retinal ganglion cells (RGCs), which form the synapses in the retinal outer and inner plexiform layers [[8](#_ENREF_8), [9](#_ENREF_9)]. Through these synapses, the visual signal is preliminarily integrated and transmitted to the brain[[10](#_ENREF_10)]. Therefore, normal synaptic structure in the retina is vital for the maintenance of the function of visual processing. Accumulating evidence has shown that changes in synapses seem to precede neuronal death after nervous injury or disease[[11](#_ENREF_11), [12](#_ENREF_12)]. Park’s and our previous study also found that the presynaptic functional protein synaptophysin (SYN) exhibited spatiotemporal alterations immediately after acute elevated IOP, which were changed before RGC death[[13](#_ENREF_13), [14](#_ENREF_14)]. However, these changes were limited to the presynaptic components without being accompanied by alterations in postsynaptic elements, which means that no new and functional synapses were formed during this process[[13](#_ENREF_13)]. As a result, these abnormal synaptic changes with vast protein synthesis and transport may lead to energy deficits of retinal neurons, which would accelerate neuron death and lead to vision deficits at last. The lack of consideration of the earlier abnormal retinal synaptic changes in the target and/or the time point of intervention for the previous rescue strategies may explain why the visual function is not effectively recovered when neural survival has been improved following many therapies. Now, the regulatory mechanisms of these synaptic changes after elevated IOP remain unclear and need to be explored.

Glia cells are the most abundant cells in the central nervous system (CNS)[[15](#_ENREF_15), [16](#_ENREF_16)]. Traditional views consider that glia cells provide substrates, energy metabolism and the physiological environment for neurons[[17](#_ENREF_17)]. In recent years, glia cells have been thought to be active modulators in developing synaptogenesis that are then silent in the developed CNS[[18](#_ENREF_18), [19](#_ENREF_19)]. In many neuropathic and nervous injury states, glia cells can be activated, characterized by increased proliferation and glial fibrillary acidic protein (GFAP) expression[[20](#_ENREF_20), [21](#_ENREF_21)]. Our previous study also demonstrated that retinal synaptic changes are accompanied by activation of macroglia cells (astrocytes and Müller cells) following acute elevated IOP[[22](#_ENREF_22)]. Meanwhile, the already increased expression of SYN was decreased when the activated macroglia cells were inhibited by fluorocitrate. This suggested that activated macroglia cells should play an important role in retinal synaptic changes induced by elevated IOP.

Several soluble factors, such as thrombospondins (TSPs), have been identified to mediate the glial influence on synaptogenesis in CNS development[[23](#_ENREF_23), [24](#_ENREF_24)]. The TSP family consists of five isoforms (TSP1/2/3/4/5) [[25](#_ENREF_25)], which are detectable in the developing CNS and then downregulated in the adult CNS [[26](#_ENREF_26)]. Most notably, TSP1 and TSP2 are the most common types in the nervous system [[20](#_ENREF_20), [27](#_ENREF_27)]. Our previous study found that the distributions of TSP1 and TSP2 in the rat retina are apparently distinct[[28](#_ENREF_28)]. TSP2 is mainly present in macroglia cells and dramatically upregulated together with increased GFAP after elevated IOP. Meanwhile, the time frame of TSP2 upregulation is consistent with the period of retinal synaptic alterations. However, TSP1 is expressed in neurons and not significantly changed with IOP. These results suggest that TSP2 is the most likely factor to be secreted by activated macroglia cells and might be involved in synaptic alterations in retinal neurons after elevated IOP. Eroglu and some researchers identified α2δ-1, a subunit of voltage-gated calcium channels, as the neuronal TSP2 receptor responsible for synaptogenesis in CNS development[[29](#_ENREF_29)]. Many other researchers also found that the expression of α2δ-1 was highly upregulated after peripheral or central nervous injury[[30](#_ENREF_30), [31](#_ENREF_31)]. Consistent with these reports, our previous study found that α2δ-1 was expressed in most retinal neurons, such as RGCs, horizontal cells, amacrine cells and bipolar cells[[28](#_ENREF_28)]. Meanwhile, the expression of α2δ-1 was increased after elevated IOP.

Based on our previous study and others’ reports, we suspect that macroglia-derived TSP2 modulates retinal alterations of presynaptic proteins by binding to its neuronal receptor α2δ-1. The results may provide new insight into novel therapeutic strategies to slow down the progress of visual function damage in retinal elevated IOP.

**Materials and Methods**

**Mixed retinal macroglia /neuron cultures (mixed cultures)**

All experimental procedures used in the present study were approved by the Institutional Review Board of the Third Xiangya Hospital of Central South University in accordance with the National Institutes of Health (NIH) guidelines for the Care and Use of Laboratory Animals. Mixed cultures of retinal macroglia/ neuron cultures were prepared from 1-day-old neonatal Sprague-Dawley rat pups as described previously[[32](#_ENREF_32)]. In brief, the eyes were removed aseptically and placed in sterile ice-cold PBS. The corneas were cut away, and the [crystalline](javascript:void(0);) lens and pigment epithelium layer were removed, leaving the retinae. The retinae were placed in Dulbecco’s modified Eagle’s medium (DMEM) containing 0.02% papain and digested in a 5% CO2 incubator at 37°C for 18 min. Subsequently, the tissue was centrifuged and transferred to a new tube containing mixed culture medium (DMEM supplemented with 10% fetal bovine serum, 5% horse serum, 1% penicillin/streptomycin and 1% L-glutamine). Then, the tissue was triturated 50 times with a Pasteur pipette and filtered with a 70-mm nylon cell strainer. The suspension was centrifuged again, and the supernatant was removed for washing the tissue. After washing, the cells were re-suspended, counted with a hemocytometer and plated in T25 flasks and 35-mm dishes at a density of 2×105 cells/ml. Cells were cultured in a 5% CO2 incubator at 37°C. Twelve hours (h) after plating, the medium was removed and replaced with fresh mixed culture medium. Every 2 days, half of the medium was changed with fresh neurobasal medium supplemented with B27 so that the concentration of the serum, which can provide energy for macroglia cells, was progressively reduced. The digital images of positively immunostained cells (in five randomly selected fields) were analyzed, and the cells were counted. The data were represented as the mean ± SEM from 3 independent experiments. The cells were used for experiments within 8-10 days after removal from the rat.

**Retinal neuron cultures**

The method is basically consistent with mixed cultures as described above with the following differences [[33](#_ENREF_33)]. First, to reach the proper neuron inoculum density, the cells were counted and plated at a density of 6×10^5^ cells/ml instead of 2×10^5^ cells/ml. Second, 4 h after plating, a time period in which the macroglia cells could not completely attach to the plates, the medium was removed so that most of the glia cells were also removed. Last, to further inhibit the proliferation of glia cells, 4 h after plating, the mixed culture medium was replaced with fresh neurobasal medium supplemented with B27. Therefore, the glia cells could not survive in this condition without serum.

**Pressure apparatus and experimental protocol**

The retinal neuron cultures or mixed cultures were exposed to elevated hydrostatic pressure (EHP) as described previously[[34](#_ENREF_34)]. The pressure variable chamber consisted of a pump, a pressure regulator, a value panel and a chamber and was placed in the normal incubator. A regulated mixture of 95% room air and 5% CO2 in the normal incubator was delivered to the chamber to achieve a constant high pressure. The pressure in the chamber was monitored by a mercurial sphygmomanometer directly connected to the outlet proximal to the chamber. The cultures were maintained under a constant high pressure of 100 mmHg for 2 h. The cells were then removed from the chamber, moved to a conventional culture incubator and allowed to recover for 2 h, 6 h, 12 h or 24 h.

**Immunofluorescence**

The cells on 35-mm dishes were fixed for 20 min with 4% paraformaldehyde (PF) and washed three times for 5 min in ice-cold 0.01 M phosphate-buffered saline (PBS) solution. Subsequently, the cells were blocked for 1 h in blocking buffer that is PBS containing 5% normal bovine serum and 0.3% Triton X-100. The cultures were then incubated with combinations of the primary antibodies against the following targets: GFAP (1:200, Calbiochem, Darmstadt, Germany), thrombospondin 2 (1:1000, Abcam, Cambridge, UK), MAP2 (1:200, Sigma-Aldrich, St. Louis, MO, USA), α2δ-1 (1:200, Sigma-Aldrich, St. Louis, MO, USA), synapsin (1:500, Synaptic Systems, Goettingen, Germany ), Homer-1b/c (1:200, SANTA, Dallas, USA), for one night at 4°C. On the next day, the cultures were shifted to room temperature for 30 min. Then, the cultures were washed 3 times as described above and incubated with Alexa-conjugated secondary antibodies (1:500, Jackson Immuno Research, West Grove, PA, USA) for 2 h. After washing three times in PBS, the cultures were covered with Vectashield mounting medium containing DAPI (Vector Laboratories, Burlingame, USA). The cells immunostained with the α2δ-1 antibody were not permeabilized with Triton-X to limit antibody staining to the cell surface[[35](#_ENREF_35)]. For immunofluorescence intensity analyses, the cell cultures were stained in parallel, and images were taken at the same setting under the fluorescence microscope.

**SiRNA approach**

To determine whether TSP2 was required for the change in synaptic proteins after EHP, we used the small interfering RNA knockdown approach to partially reduce TSP2 expression. The siRNA kit against TSP2 was obtained from RIBO-Biology (Guang Zhou, China), and the transfection reagent was from MACS (Teterow, Germany). In companion control experiments, glia cells were transfected with the same amount of either a targeting control or a non-targeting control. Another group with transfection reagent alone was used as a normal control. The transfection protocol followed the MACS manufacturer’s instructions: First, the siRNA and transfection reagent were separately diluted in opti-MEM (Gibco, Grand Island, USA) for 5 min. Then, the diluted siRNA and transfection reagent solutions were mixed together for another 20 min for complex formation. Last, the cell medium was replaced with neurobasal medium, and the transfection complexes were added dropwise to the cultures. Four hours after transfection, the culture medium was replaced with normal neurobasal medium plus B27. Twenty-four hours after transfection, the cultures were collected for the next experiments. Knockdown of TSP2 was validated by Western blot.

**Drug application**

Purified human recombinant TSP2 protein was purchased from R&D systems (Minnesota, USA) and gabapentin (GBP) from SANTA CRUZ (Dallas, USA). All drugs were dissolved in sterile PBS as stock solutions and warmed to room temperature before performing experiments. TSP2, which was used at a concentration of 10 nM (~1.29 μg/ml), and gabapentin, which was used at a concentration of 32 μM (~5.47 μg/ml), were administered directly to cell cultures before high pressure treatment. The incubations were maintained for 14 h, including 2 h cultured in high pressure, and subsequently subjected to immunofluorescence and Western Blot.

**Statistical analysis**

Figure panels were assembled by using Photoshop CC. The measurement data were presented as the mean ± SD. One-way analysis of variance and independent sample t-tests were used to analyze the data using GraphPad Prism 5 software (GraphPad Software Inc, San Diego, CA, USA). A value of P < 0.05 was considered statistically significant. The statistical graphs were created with GraphPad Prism 5 software.

**Results**

**Culture compositions**

The culture compositions were determined by cell counting after double immunofluorescence staining. Map2 and GFAP were used as markers of neurons and macroglia, respectively, and the cell nuclei were labeled with DAPI. The results showed that the mixed cultures consisted of approximately (33.16±2.56) % neurons and (60.15±2.1) 6% macroglia cells (Fig 1A). The retinal neuron cultures appeared to be (92.35±1.68) % pure as assessed by immunocytochemistry of Map2 (Fig 1B).

**Fig 1. Cell compositions in the mixed cultures and retinal neuron cultures.** (A) Double immunofluorescence Map2/GFAP staining in mixed cultures. (B) Double immunofluorescence Map2/DAPI staining in retinal neuron cultures. Scale bar=50 μm.

**Localization of TSP2 and α2δ-1**

Double immunofluorescence staining was performed, and TSP2 staining was co-localized with GFAP immunoreactivity (Fig 2A). α2δ-1 was strongly expressed in the retinal neuron bodies, and reduced staining was observed in dendrites visualized by co-localization with Map2, which is consistent with Farrell’s report[[36](#_ENREF_36), [37](#_ENREF_37)] (Fig 2B).

**Fig 2. Immunofluorescence for the localization of TSP2 and α2δ-1.** (A) Localization of TSP2 was identified by TSP2/GFAP co-staining. (B) Localization of α2δ-1 was identified by α2δ-1/Map2 co-staining. Scale bar=50 μm.

**The expression levels of presynaptic proteins and GFAP were increased after EHP in mixed cultures**

First, we quantified the expression levels of the pre/postsynaptic proteins using Western Blot and immunofluorescence in the EHP groups (recovery time point for each group: 2 h, 6 h, 12 h and 24 h) after 2 h EHP in mixed cultures. In the immunofluorescence assay, no difference in the distribution of synapsin and Homer was observed between the injury groups and the control group. Meanwhile, the immunofluorescence intensity of synapsin in the EHP groups was higher (Fig 3A) than in the control group, but the immunofluorescence intensity of Homer was not changed (Fig 3A). From the results of the Western blot, statistical analysis of the integrated density value (IDV) indicated that the expression levels of presynaptic SYN and synapsin were significantly increased 6 h, 12 h and 24 h (P＜0.01) after EHP (Fig 3B and 3C). However, exposure of mixed cultures to EHP had no effect on the expression of postsynaptic PSD95 and Homer (Fig 3B and 3C). These results showed that, in mixed cultures, EHP could increase the expression of presynaptic SYN and synapsin but not postsynaptic PSD95 and Homer.

Meanwhile, we observed a continuous increase in reactive macroglia cells indicated by increased proliferation and GFAP expression (Fig 3A). Statistical analysis of the Western blot results showed that the expression of GFAP was significantly increased 6 h (P＜0.05), 12 h (P＜0.005) and 24 h (P＜0.005) after EHP (Fig 3B and 3C). Together, this and the above results indicated that retinal macroglia cells were activated when the expression of retinal presynaptic proteins was changed following EHP.

**Fig 3. Expression of GFAP, SYN, synapsin, PSD95 and Homer following EHP in mixed cultures.** Labels are as follows: Control group (Control); 2, 6, 12, and 24 h after EHP (2 h, 6 h, 12 h, and 24 h). (A) Immunofluorescence staining of GFAP, synapsin and Homer following EHP. The lower panels are the magnified images of the area in the rectangles of the upper panels. (B) Western blot of GFAP, SYN, synapsin, PSD95 and Homer following EHP. (C) The statistical analysis of GFAP, SYN, synapsin expression by Western blot following EHP, * compared to Control, P＜0.05; ** compared to Control, P＜0.01; *** compared to Control, P＜0.005. PSD95 and Homer expression show no statistically significant difference among groups (P>0.05). Scale bar=50 μm.

**The expression levels of synaptic proteins were not changed after EHP in retinal neuron cultures**

To investigate whether macroglia cells are necessary for the alterations in the presynaptic proteins after EHP, retinal neurons were cultured to eliminate the influence of macroglia cells. Retinal neuron cultures were exposed to EHP as described before, and no distinct changes in the density of pre/postsynaptic protein puncta were observed between the EHP groups and control group (Fig 4A). Statistical analysis of the Western blot results indicated that EHP did not significantly change the expression of pre/postsynaptic proteins (Fig 4B and 4C). These results indicated that, in retinal neuron cultures without macroglia cells, EHP could not change the expression of synaptic proteins. Taken together, these results further suggested that macroglia cells may be involved in the change in retinal synapses following EHP.

**Fig 4. Expression of SYN, synapsin, PSD95 and Homer following EHP in retinal neuron cultures.** Labels are as follows: Control group (Control); 2, 6, 12, and 24 h after EHP (2 h, 6 h, 12 h, and 24 h). (A) Immunofluorescence staining of synapsin and Homer following EHP. The lower panels are the magnified images of the area in the rectangles of the upper panels. (B) Western blot of SYN, synapsin, PSD95 and Homer following EHP. (C) The statistical analysis of SYN, synapsin, PSD95 and Homer protein expression following EHP, showing no statistically significant difference among groups (P>0.05). Scale bar=50 μm.

**Upregulation of TSP2 and presynaptic proteins after EHP was downregulated in cultures treated with TSP2 siRNA**

Mixed cultures were exposed to EHP as described before, and the immunofluorescence intensity of TSP2 was enhanced in the injury groups compared with that in the control group (Fig 5A). Western Blot results indicated that EHP significantly increased the expression of TSP2 6 h, 12 h and 24 h after EHP (P＜0.01) (Fig 5B and 5C).

The synaptogenic properties of TSP2 and the relative increase in TSP2 and presynaptic proteins after EHP prompted us to research whether TSP2 is required for the upregulation of presynaptic proteins after EHP. To solve this, we examined whether silencing TSP2 with siRNA could prevent or diminish the level of upregulation of presynaptic or postsynaptic proteins. In the experiments above, we found that the expression of presynaptic proteins and TSP2 peaked at 6 h, 12 h, and 24 h after EHP, and we chose the middle time point (12 h) as our primary intervention time point. First, we designed an artificial siRNA targeting TSP2 and transfected it into the mixed cultures. TSP2 was knocked down in the mixed cultures with the siRNA as demonstrated by the weaker immunoreactivity (Fig 6A) and statistical analysis of the Western Blot result (P＜0.005) (Fig 6B and 6C). Then, in contrast with that in the 12-h EHP group, a weaker staining of synapsin was found in the siRNA group (Fig 6A). The Western blot results also showed that the expression levels of SYN (P＜0.01) and synapsin (P＜0.005) in the siRNA group were lower than in the 12-h group (Fig 6B and 6C). Last, we examined whether decreased TSP2 expression could lead to downregulation of PSD95 and Homer. Conversely, the decreased expression of TSP2 did not cause any changes in the expression of postsynaptic proteins as shown by the immunofluorescence and Western Blot assay (Fig 6A, 6B and 6C). Taken together, these results indicate that TSP2 is essential for the upregulation of presynaptic proteins in retinal neurons after EHP.

**Fig 5. Expression of TSP2 under EHP.** Labels are as follows: Control group (Control); 2, 6, 12, and 24 h after EHP (2 h, 6 h, 12 h, and 24 h). (A) Immunofluorescence staining of TSP2 following EHP. The lower panels are the magnified images of the area in the rectangles of the upper panels. (B) Western blot of TSP2 expression following EHP. (C) Statistical analysis of TSP2 expression following EHP, ** compared to Control, P＜0.01. Scale bar=50 μm.

**Fig 6. Expression of TSP2, SYN, synapsin, PSD95 and Homer under EHP and TSP2 siRNA silencing.** Labels are as follows: Control group (Control), 12 h after EHP (12 h), Reagent + 12 h after EHP (12 h + Reagent), Non-targeting control + 12 h after EHP (12 h + NControl), siRNA + 12 h after EHP (12 h + siRNA). (A) Immunofluorescence staining of TSP2, synapsin and Homer following EHP and siRNA knockdown. The lower panels are the magnified images of the area in the rectangles of the upper panels. (B) Western blot of TSP2, SYN, synapsin, PSD95 and Homer expression following EHP. (C) The statistical analysis of TSP2, SYN and synapsin expression following EHP and TSP2 siRNA, * compared to Control, P＜0.05. PSD95 and Homer expression show no statistically significant among groups (P>0.05). Scale bar=50 μm.

**Recombinant TSP2 protein upregulated the levels of presynaptic proteins after EHP in retinal neuron cultures**

We also examined whether recombinant TSP2 protein directly contributed to the upregulation of the levels of presynaptic proteins after EHP. Accordingly, we applied recombinant TSP2 protein to retinal neuron cultures (without astrocytes; all TSP2 comes from the intervention) at the beginning of exposure to high pressure. The analysis was also conducted 12 h after EHP. Direct exposure of retinal neurons to recombinant TSP2 protein for 12 h after EHP (TSP2 group) resulted in a higher density of synapsin-positive puncta than in the normal control group but did not change the density of Homer (Fig 7A). The Western blot results also corresponded well with the findings in the immunofluorescence assay showing that the expression levels of SYN (P＜0.05) and synapsin (P＜0.001) in the TSP2 group were higher than in the 12-h group, while the expression levels of PSD95 and Homer were maintained with or without treatment (Fig 7B and 7C). These data provided further evidence that TSP2 could directly upregulate the levels of presynaptic proteins after EHP in retinal neurons.

**Fig 7. Expression of SYN, synapsin, PSD95 and Homer under EHP and with the addition of recombinant TSP2 protein in retinal neuron cultures.** Labels are as follows: Control group (Control), 12 h after EHP (12 h), recombinant TSP2 protein + 12 h after EHP (12 h + TSP2). (A) Immunofluorescence staining of synapsin and Homer proteins following EHP and the addition of recombinant TSP2 protein. The lower panels are the magnified images of the area in the rectangles of the upper panels. (B) Western blot of SYN, synapsin, PSD95 and Homer expression under EHP with the addition of recombinant TSP2 protein. (C) The statistical analysis of SYN and synapsin proteins following EHP and the addition of recombinant TSP2 protein, * compared to Control，P＜0.05. PSD95 and Homer expression show no statistically significant difference among groups (P>0.05). Scale bar=50 μm.

**Upregulated α2δ-1 was involved in synaptic changes after EHP in mixed cultures**

In this experiment, we investigated whether TSP2 modulated the presynaptic alterations of retinal neurons following EHP by binding to α2δ-1. Mixed cultures were exposed to EHP as described before. The immunofluorescence intensity of α2δ-1 was significantly enhanced after EHP compared with that in the normal control group (Fig 8A). The Western blot results indicated that the expression levels of α2δ-1 6 h, 12 h and 24 h after EHP were increased compared with that in the control group (P＜0.05) (Fig 8B and 8C).

Then, we used GBP to block the interaction of α2δ-1 with TSP2. GBP was added to the mixed cultures at the beginning of EHP, and the mixed cultures with GBP were incubated for another 12 h after EHP. We first tested whether GBP could decrease the expression of α2δ-1 on the cell surface. As was shown in the immunofluorescence assay, the immunofluorescence intensity of α2δ-1 in the GBP group was weaker than in the 12-h group (Fig 9A). This indicated that GBP could effectively decease the cell surface expression of α2δ-1 so that GBP could block the interaction of TSP2 with α2δ-1[[35](#_ENREF_35)].

In the next experiment, we verified whether cell-surface α2δ-1 muting could reverse the upregulation of presynaptic proteins induced by EHP. After GBP treatment, weaker labeling of synapsin was found in the GBP group (Fig 9A), but the staining of Homer remained unchanged (Fig 9A). The statistical analysis of the Western blot results indicated that GBP treatment decreased the expression of SYN and synapsin in the GBP group compared with that in the 12-h group but did not change the expression of PSD95 and Homer (Fig 9B and 9C). These results suggested that GBP could decrease the production of presynaptic proteins by blocking the interaction of α2δ-1 with TSP2 in mixed cultures.

**Fig 8. Expression of α2δ-1 under EHP.** Labels are as follows: Control group (Control); 2, 6, 12, and 24 h after EHP (2 h, 6 h, 12 h, and 24 h). (A) Immunofluorescence staining of α2δ-1 following EHP. The lower panels are the magnified images of the area in the rectangles of the upper panels. (B) Western blot of α2δ-1 expression under EHP. (C) Statistical analysis of α2δ-1 expression levels under EHP, * compared to Control, P＜0.05. Scale bar=50 μm.

**Fig 9. Expression of α2δ-1 under EHP and GBP treatment in mixed cultures.** Labels are as follows: Control group (Control), 12 h after EHP (12 h), GBP + 12 h after EHP (12 h + GBP). (A) Immunofluorescence staining of α2δ-1, SYN, synapsin, PSD95 and Homer following EHP and GBP treatment. The lower panels are the magnified images of the area in the rectangles of the upper panels. (B) Western blot of SYN, synapsin, PSD95 and Homer expression under EHP and GBP treatment. (C) Statistical analysis of SYN and synapsin expression levels under EHP and GBP treatment, * compared to Control, P＜0.05. PSD95 and Homer expression show no statistically significant difference among groups (P>0.05). Scale bar=50 μm.

**Discussion**

Many reports have demonstrated that acute or continuous elevation of IOP could lead to retinal neuron death and visual deficits[[1](#_ENREF_1)]. Cultured cells exposed to elevated pressure are commonly used in neuronal death models induced by elevation of IOP[[38](#_ENREF_38)]. In our study, the open cycling air pressure culture system, which could set defined pressure values as required, was applied to induce high pressure and injure the cultured cells to mimic elevated IOP injury. Meanwhile, we used SYN and synapsin[[26](#_ENREF_26), [39](#_ENREF_39)], presynaptic functional proteins that are widely used as markers of synaptic plasticity, as well as PSD95 and Homer[[39](#_ENREF_39), [40](#_ENREF_40)], the popular markers of postsynaptic density, to observe synaptic changes in retinal neurons. We found that the expression levels of presynaptic SYN and synapsin in mixed cultures were increased after EHP. However, EHP did not change the expression of postsynaptic PSD95 and Homer. We also found that GFAP immunoreactivity was increased, which indicated that macroglia cells were activated together with the presynaptic changes after EHP. At the same time, TSP2 and α2δ-1 protein levels also increased in mixed cultures after exposure to EHP. Thus, activated macroglia cells and the expression change of TSP2, α2δ-1 and presynaptic proteins could be triggered in concert with an associated event.

Glia cells have been shown to be able to be activated in neuropathic conditions, and the activated glia cells may release reactive factors to promote synaptogenesis in the developed nervous system. Then, the reactive synaptogenesis may result in various nervous system diseases [[15](#_ENREF_15), [20](#_ENREF_20), [21](#_ENREF_21)]. Lo[[41](#_ENREF_41)] found that, after neonatal unilateral transection of the infraorbital branch of the trigeminal nerve, glia cells were activated, demonstrated by cell proliferation and upregulation of GFAP, and accompanied by reactive synaptogenesis in the nerve projection zone. Meanwhile, reactive synaptogenesis was blocked when sodium fluoroacetate blocked the activated glia cell function. Consistent with this report, we found that retinal macroglia cells were activated after EHP in mixed cultures. Meanwhile, the expression levels of presynaptic SYN and synapsin in mixed cultures were increased, but the expression levels of postsynaptic proteins were not changed. Next, we removed the macroglia cells from the mixed cultures and found that retinal neuron cultures exposed to EHP did not exhibit a change in the expression of these presynaptic proteins. This was consistent with Crawford’s report that glia cell deprivation with 4% paraformaldehyde/0.2% glutaraldehyde mediated hippocampal presynaptic plasticity[[42](#_ENREF_42)]. That result indicates that the increased expression of presynaptic proteins, without the accompanying changes in the expression of postsynaptic proteins, cannot form intact and functional synapses after EHP. Taken together, these results indicated that the activated macroglia cells play important roles in the changing process of synaptic proteins in retinal neurons induced by EHP. Now, it is possible to consider therapeutic strategies that target macroglia cells for the treatment of diseases induced by EHP. However, inhibition of macroglia cells, which play important roles in the nervous system, could also lead to other neurodegenerative diseases and injuries. Therefore, it is reasonable and important to discover the mechanisms of presynaptic plasticity induced by activated macroglia cells after EHP.

TSPs have been proven to be a key factor secreted by glia cells to promote synapse formation in the developing central and peripheral nervous system[[24](#_ENREF_24), [39](#_ENREF_39)]. Further research has demonstrated that following some nervous diseases or injury, such as stroke, hepatic encephalopathy, cerebral ischemia and hemorrhage, glia cells can be activated and re-express TSPs, which participate in the regulation of neuronal plasticity[[43-45](#_ENREF_43)]. Crosby[[46](#_ENREF_46)] found that increased expression of TSPs in the spinal cord following injury contributes to abnormal synaptogenesis and leads to neuropathic pain. Blocking TSP expression with antisense oligonucleotides can inhibit dorsal horn synaptogenesis and then attenuate injury-induced allodynia. In our prior study, we found that TSP2 was mainly located in retinal macroglia cells and sharply increased in vivo after EHP. The distribution and expression change of the TSP2 protein suggested that TSP2 might be the key molecule secreted by macroglia cells that may modulate synaptic changes after EHP. To prove the speculation above, we first utilized siRNA to silence TSP2 expression at the time when its protein level was upregulated by EHP in mixed cultures. In accordance with our prediction, we observed a significant decrease in both SYN and synapsin expression. To directly confirm that the TSP2 protein has an effect on the change in presynaptic protein expression after EHP, we added recombinant TSP2 protein to retinal neuron cultures. We found that exposure of neurons to the TSP2 protein could lead to increased expression of SYN and synapsin proteins. These findings strongly indicated that TSP2 may modulate presynaptic protein changes in retinal neurons following EHP. These results suggest that suppressing the upregulation of TSP2 may be a good way to inhibit the change in presynaptic proteins induced by EHP.

Eroglu identified that TSPs promote synapse formation by interacting with the neuronal receptor α2δ-1[[29](#_ENREF_29)]. Andresen[[47](#_ENREF_47)] also found that in an experimental model, following neonatal freeze-lesion, the expression levels of both TSP and α2δ-1 were transiently increased, resulting in synaptogenesis and cortical hyperexcitability. Treatment with GBP, a widely used clinical pharmaceutical drug that is used for treatment of pain, anxiety and epilepsy, could disrupt the interaction of TSP with α2δ-1 and would prevent the formation of epileptic activity[[30](#_ENREF_30), [48](#_ENREF_48)]. Taken together, we hypothesized that α2δ-1 may be the receptor of TSP2 involved in the presynaptic alterations in retinal neurons following EHP. Meanwhile, treatment with GBP might be one way to inhibit the significant change in presynaptic protein expression in the retina after elevated IOP. Therefore, in this study, we discovered that α2δ-1 was localized to retinal neurons (strongly immunostained in soma and weakly immunostained in dendrites) in vitro, which was the same in vivo. Then, in accordance with others reports, we found a significant increase in α2δ-1 in immunoreactivity after EHP, which coincided with TSP2 upregulation and presynaptic changes, suggesting that α2δ-1 may contribute to the changes in retinal synaptic plasticity after EHP. Next, we found that pretreatment with GBP, the inhibitor of the interaction of TSP2 with α2δ-1, could reduce the increase in presynaptic SYN and synapsin induced by EHP. Thus, our results indicated that α2δ-1 may be the receptor for TSP2 involved in the retinal synaptic changes after EHP. Furthermore, using GBP may contribute to the prevention of the significant change in presynaptic protein expression by inhibiting the interaction of TSP2 with α2δ-1 after EHP.

In the current study, we observed the upregulation of presynaptic proteins without the accompanying changes in postsynaptic proteins in primary mixed cultures exposed to EHP for 2 h. That means that EHP only induced presynaptic protein changes, but no intact and functional synapses were formed[[13](#_ENREF_13), [22](#_ENREF_22)]. However, protein synthesis function is highly dependent on mitochondrial energy production, and abnormal synthesis function may lead to energy deficits in cells and increased apoptosis[[49](#_ENREF_49)]. Therefore, this presynaptic change process may place a burden on the energy-producing machinery of neurons and accelerate the processes of synaptic disruption and neuronal death[[43](#_ENREF_43), [50](#_ENREF_50)]. Studying this regulatory mechanism of presynaptic changes and looking for targets to inhibit this process may be able to save this ineffective energy consumption and eventually lead to new ideas for retinal protection. The current experiments first confirmed that retinal microglia activation after EHP could modulate these synaptic alterations in retinal neurons and, next, determined that TSP2 secreted by macroglia cells in the presence of environmental stress induced during the course of EHP might regulate synaptic protein alterations by binding to its neuronal receptor α2δ-1 in primary mixed cultures. This regulatory pathway may represent attractive targets for pharmacological intervention and require the profound understanding of their distribution and role in the nervous system to develop targeted therapies for diseases and injuries[[51](#_ENREF_51)]. Therefore, it is reasonable to expect that strategies targeting the macroglia-TSP2-α2δ-1 pathway to regulate presynaptic changes of retinal neurons after EHP may contribute to the recovery of visual function. Also, we suspect that another way to protect the visual pathway is to stimulate postsynaptic changes, such as those induced by glypicans, which can arouse the response of the postsynaptic components in response to the presynaptic synaptogenesis events[^48,49^](#_ENREF_48). One possibility is that the complementary mechanism of postsynaptic plasticity of retinal neurons is integral for the modulatory role of the macroglia cells after EHP, which requires further experimental exploration.

**Conclusion**

This study demonstrated that retinal microglia cells were activated in vitro after EHP, resulting in presynaptic alterations without the accompanying changes in postsynaptic proteins in retinal neurons. We next determined that TSP2 secreted by macroglia cells after EHP injury might regulate the presynaptic protein alteration by binding to its neuronal receptor α2δ-1. These results suggest that targeting the macroglia cell-TSP2-α2δ-1 pathway may contribute to the recovery of visual function after elevated IOP.
